# Conducting polyfurans by electropolymerization of oligofurans†
†This work is dedicated to the memory of Professor Michael Bendikov.
‡
‡Electronic supplementary information (ESI) available: Full experimental and computational details, synthesis of **1–18**, Fig. S1–S26 and Tables S1–S4. See DOI: 10.1039/c4sc02664k



**DOI:** 10.1039/c4sc02664k

**Published:** 2014-10-17

**Authors:** Dennis Sheberla, Snehangshu Patra, Yair H. Wijsboom, Sagar Sharma, Yana Sheynin, Abd-Elrazek Haj-Yahia, Adva Hayoun Barak, Ori Gidron, Michael Bendikov

**Affiliations:** a Department of Organic Chemistry , Weizmann Institute of Science , Rehovot , 76100 , Israel

## Abstract

Polyfurans have never been established as useful conjugated polymers, as previously they were considered to be inherently unstable and poorly conductive. Here, we show the preparation of stable and conducting polyfuran films by electropolymerization of a series of oligofurans of different chain lengths substituted with alkyl groups. The polyfuran films show good conductivity in the order of 1 S cm^–1^, good environmental and electrochemical stabilities, very smooth morphologies (roughness 1–5 nm), long effective conjugation lengths, well-defined spectroelectrochemistry and electro-optical switching (in the Vis-NIR region), and have optical band-gaps in the range of 2.2–2.3 eV. A low oxidation potential needed for polymerization of oligofurans (compared to furan) is a key factor in achievement of improved properties of polyfurans reported in this work. DFT calculations and experiments show that polyfurans are much more rigid than polythiophenes, and alkyl substitution does not disturb backbone planarity and conjugation. The obtained properties of polyfuran films are similar or superior to the properties of electrochemically prepared poly(oligothiophene)s under similar conditions.

## Abstract 2

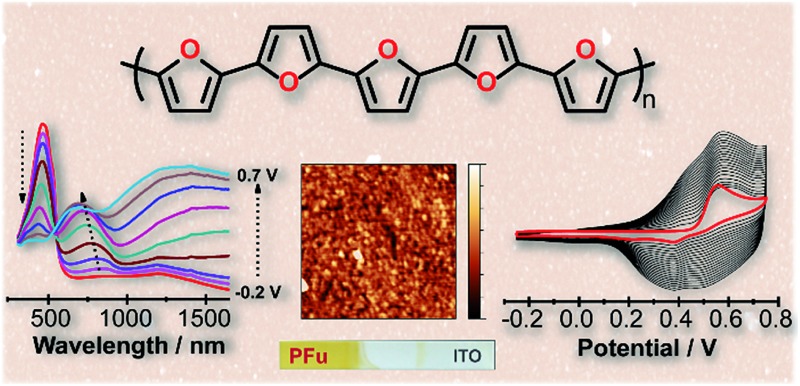
Polyfuran films produced by electropolymerization of a series of oligofurans substituted with alkyl groups show improved properties, such as good conductivity and stability, well-defined spectroelectrochemistry and smooth morphology.

## Introduction

The field of conjugated and electrically conducting polymers continues to attract much attention both scientifically and technologically.^1^ Among them, an important class of five-membered unsaturated heterocyclic polymers, such as polypyrrole (PPy),^2^ polythiophene (PT),^3^ polyselenophene (PSe)^4^ as well as their derivatives, have been extensively studied and applied. Polyfuran (PFu), on the other hand, has scarcely been explored and has not been established as a conductive polymer. This is because of the difficulty in polymerization, the low stability and the poor properties of the polyfuran samples that have been obtained.^5^ The reported electrical conductivities of doped polyfurans were measured in the range of 10^–5^ to 10^–2^ S cm^–1^,^5,6^ which is significantly lower than the conductivity of polythiophenes electrochemically prepared from a terthiophene (PT_3_) (10^0^ S cm^–1^)^3b,7^ and thiophene monomers (10^2^ S cm^–1^).^3b,8^ Polyfurans have also been reported as environmentally and electrochemically unstable. For example, the authors noticed that upon exposure to ambient light and air, films of chemically prepared alkylated polyfuran bleached over a period of hours to days.^9^ Furthermore, the electrochemical redox activity of polyfuran was shown to degrade during cycling in acetonitrile (ACN) solution.^10^

Many researchers have tried to overcome preparation problems by varying the conditions of polymerization.^5,11^ The main problem associated with electrochemical polymerization is the high oxidation potential required to polymerize furan, which results in irreversible over-oxidation of the resulting polyfurans.^5,12^ A mixture of trifluoride diethyl etherate and ethyl ether (BFEE/EE) as a solvent was used to reduce this electropolymerization potential.^11c,d,13^ However, the reported conductivities of the resulting polyfurans were small (up to 10^–2^ S cm^–1^), and their absorption spectra (*λ*_max_ = 420 nm)^11*d*^ reveal a short conjugation length of about eight furan rings.^14^ Kanatzidis *et al.*, used terfuran as the starting monomer to lower the potential needed for polymerization to less than 1.0 V (*vs.* SCE).^11*b*^ The resulting polyfuran was characterized by a variety of techniques and showed a red shifted absorbance maximum centered at 468 nm. Nevertheless, the polyfuran produced had a low conductivity of 2 × 10^–3^ S cm^–1^, which is probably due to defects in the polymer, as were observed by IR spectroscopy. Chemically synthesized polyfuran did not appear to be highly conjugated because of a significant degree of furan ring-opening defects.^9,15^ It is noteworthy that in contrast to polyfurans, the incorporation of furan blocks in conjugated polymers resulted in high quality materials.^16^ Overall, despite numerous reports on the preparation of polyfuran (usually lacking full characterization),^5^ it appears that there are no reports of polyfurans with properties such as high conductivity, stability, high conjugation length and small number of defects, all of which are required for their application as organic electronic materials.

We previously reported the preparation of long unsubstituted α-oligofurans (Fu_5_-Fu_9_) and recently alkyl substituted α-oligofurans (Fu_*n*_-2C_6_ (*n* = 4, 6, 8) and Fu_16_-6C_6_) that exhibit higher fluorescence, better packing, greater rigidity, and higher solubility than the corresponding oligothiophenes.^14,17^ We also showed that these oligofurans display good electronic properties, *e.g.*, field effect mobilities and on-off ratios, similar to those of the corresponding oligothiophene analogues.^18^ Thus, we surmised that polyfurans would not only possess similar properties, but would have advantages over other conductive polymers. The rigidity of their backbone is expected to enable the introduction of large side substituents without breaking planarity and π-conjugation. Moreover, polyfurans can be synthesized from renewable resources^19^ and may be biodegradable.^20^ Thus, we believe that the reported poor properties, such as low conductivity,^5^ blue-shifted absorption,^11*d*^ and instability,^9^ of polyfurans were due to defects produced during their preparation rather than intrinsic properties of polyfurans.

**Chart 1:**
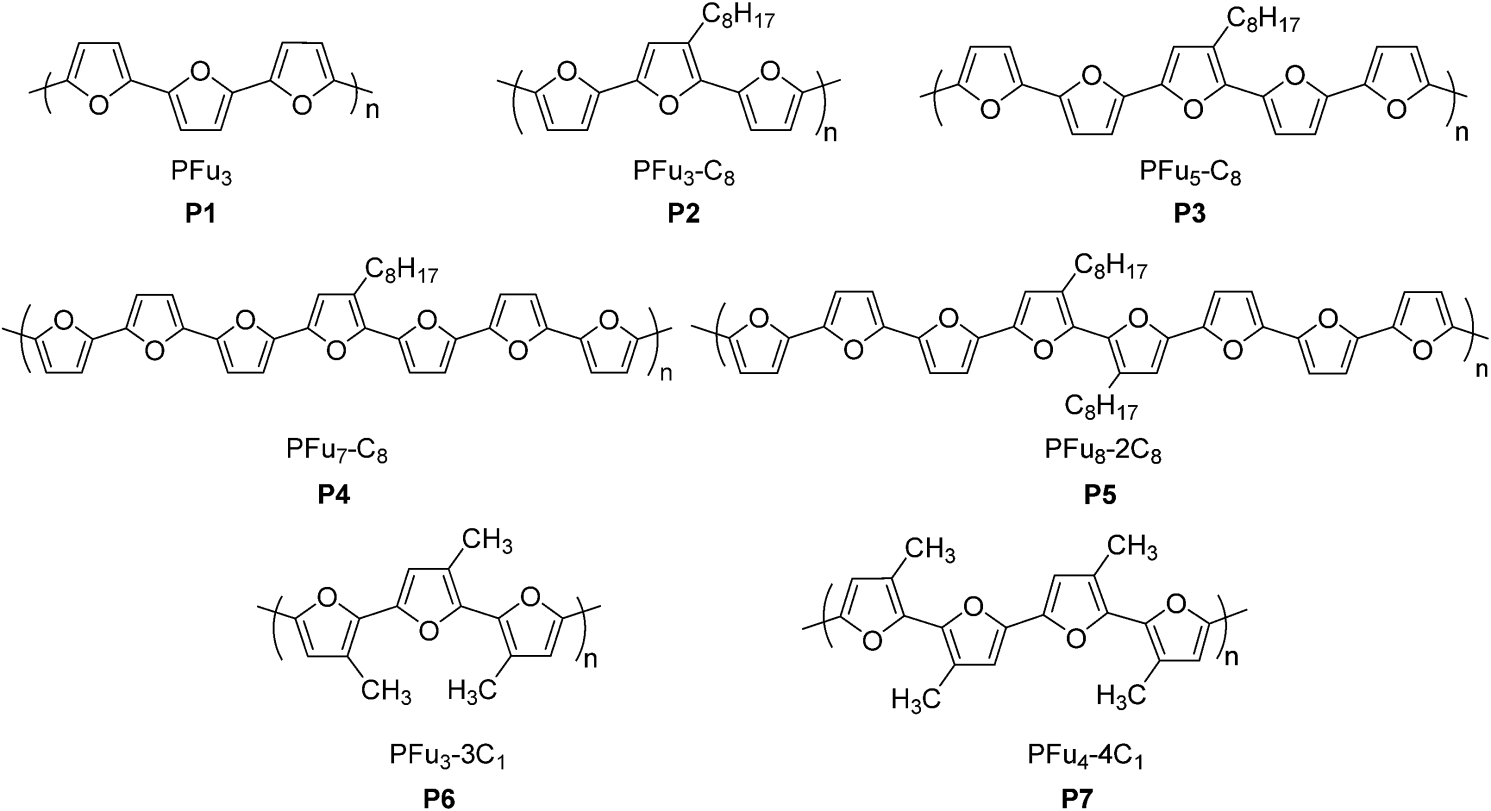
Polymers prepared and studied in this work.

Here we report the first electrochemically-prepared stable and conducting polyfuran films **P1–P7** (Chart 1), which possess all the properties necessary to be considered as conducting polymers suitable for applications in organic electronics. These polymers have several advantages over other conjugated polymers, such as rigidity of the backbone and film smoothness. The use of long oligofurans with solubilizing groups, which have relatively low oxidation potential, as the starting monomers for polymerization was the key factor in obtaining polyfuran with improved properties. The electrochemical and spectroelectrochemical behavior, conductivity, electrochemical stability in air under ambient conditions, as well as the morphology of the prepared polyfuran films were studied and compared with corresponding polythiophene analogues. We also report the development of a synthetic methodology for the preparation of long oligofurans **1–7** substituted with solubilizing groups.

## Results

### Synthesis of oligofurans

**Scheme 1:**
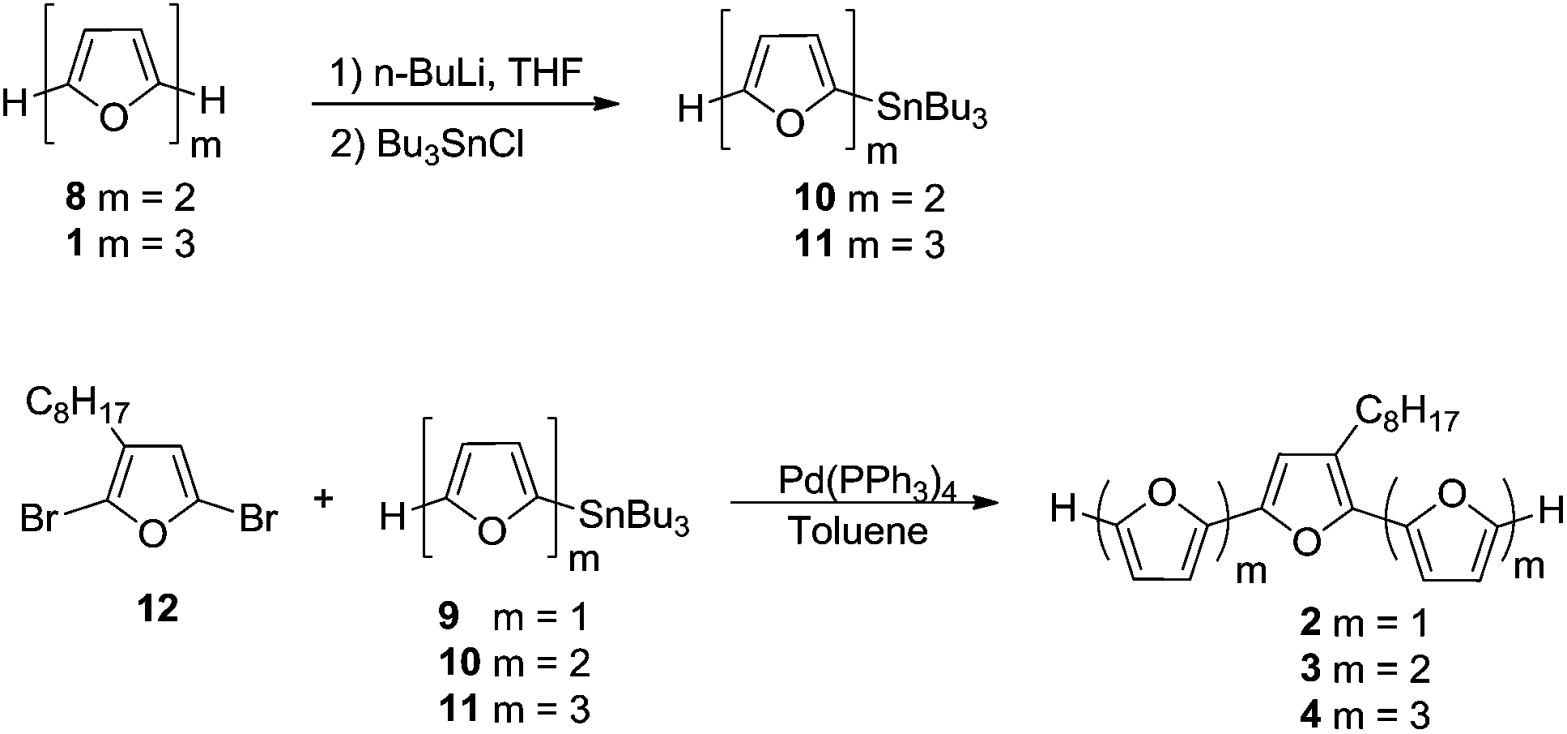


**Scheme 2:**
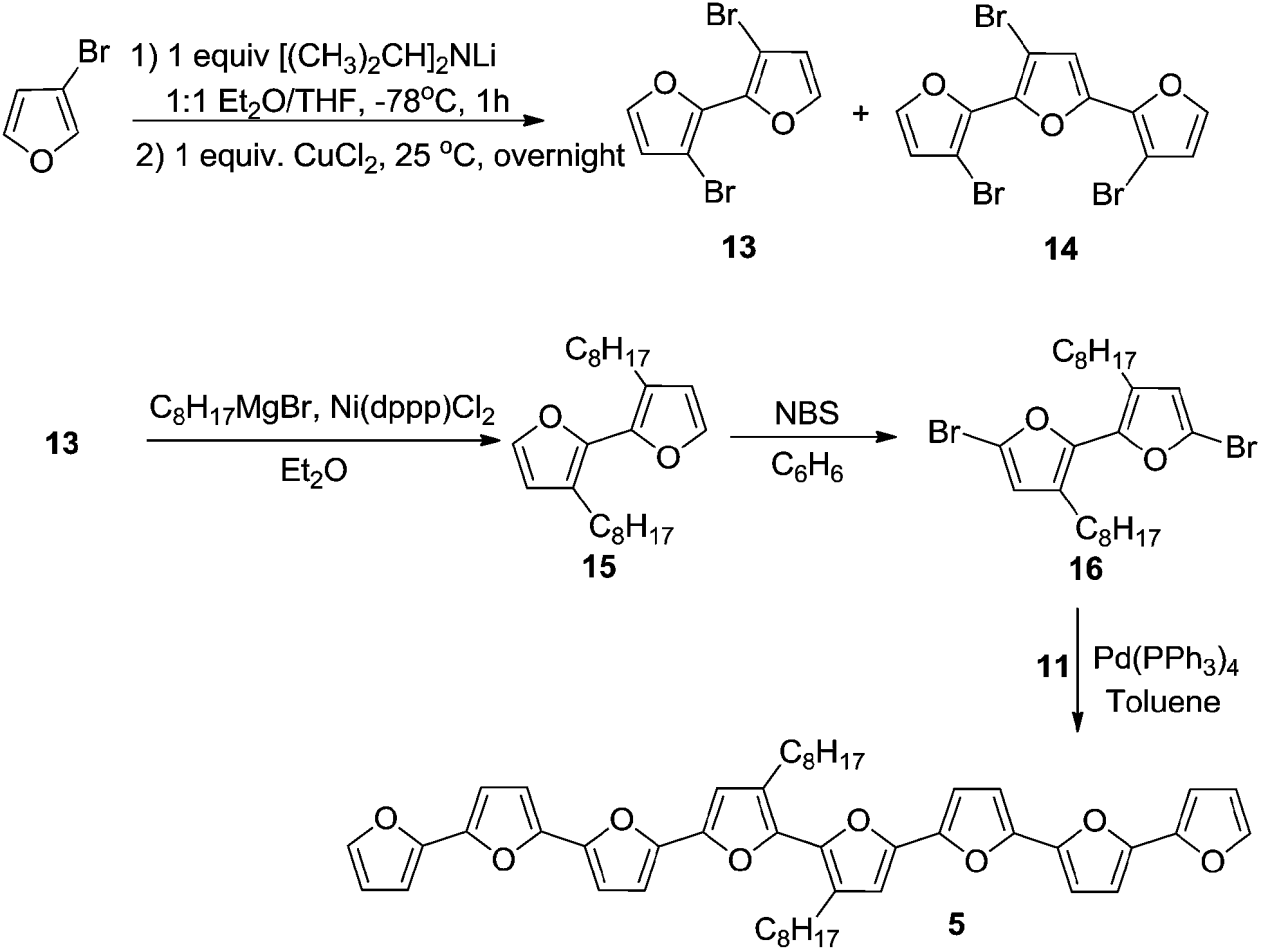


**Scheme 3:**
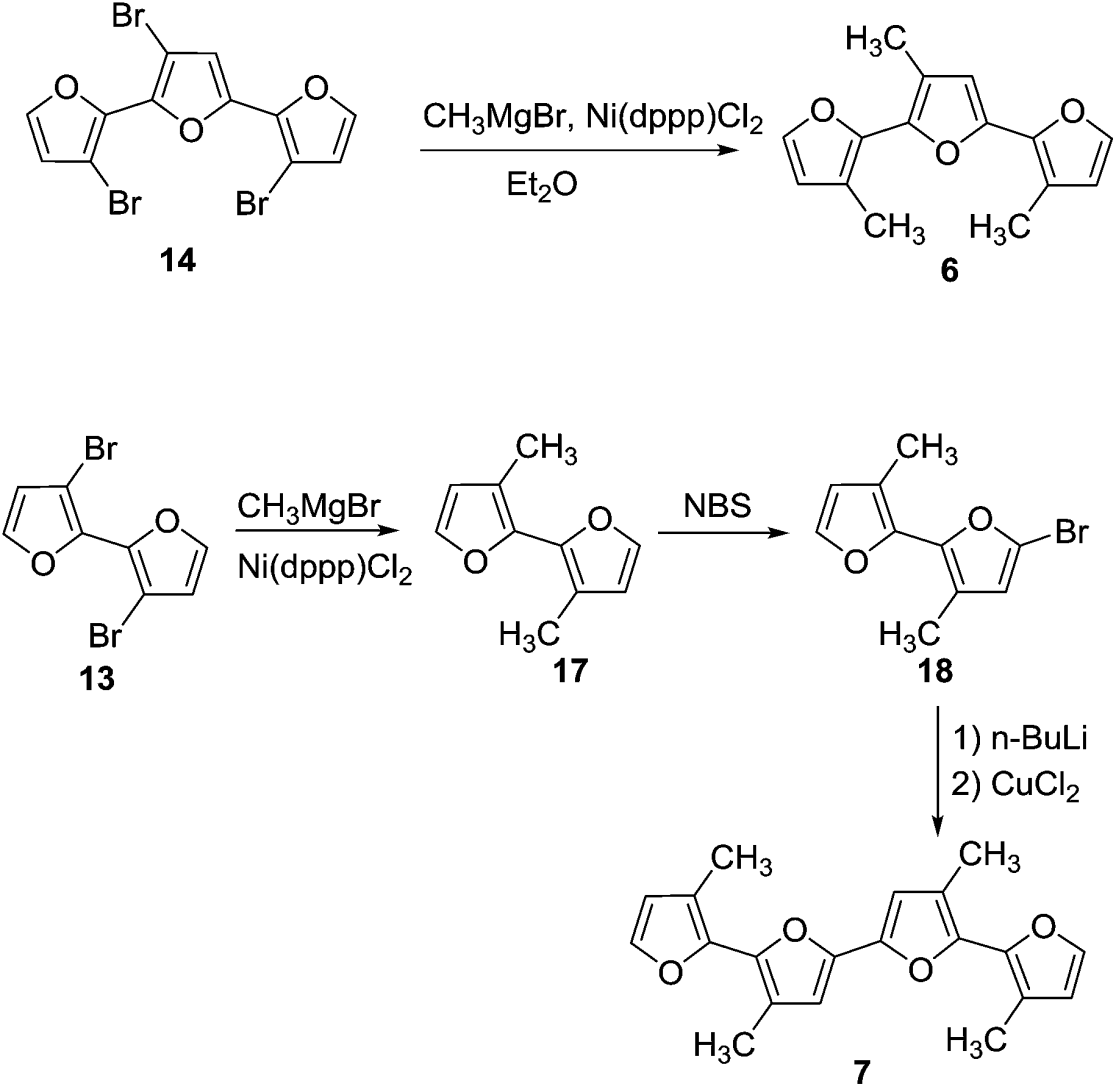


Oligomers **1–5** (Schemes 1 and 2) were synthesized by Stille coupling, oligomer **6** (Scheme 3) was synthesized by Kumada coupling, and oligomer **7** (Scheme 3) was obtained by a combination of Kumada and Ullmann couplings of suitable precursors. Synthesis of compounds **1**, **10** and **11** was reported previously.^14,21^ Synthesis of oligomers **2**, **3**, and **4** (Scheme 1) was achieved by Stille coupling of 2,5-dibromo-3-octylfuran (**12**) with tributyl(furan-2-yl)stannane (**9**), [2,2′-bifuran]-5-yltributylstannane (**10**), and [2,2′:5′,2′′-terfuran]-5-yltributylstannane (**11**), respectively. The precursor 2,5-dibromo-3-octylfuran (**12**) was prepared by bromination of 3-octylfuran with *N*-bromosuccinimide (NBS) with a 68% yield.

For the synthesis of octafuran **5** (Scheme 2), dibromo derivative **13** was prepared using the procedure described in the literature.^22^ Interestingly, during the course of the reaction, terfuran derivative **14** was also obtained with a 6% yield. Subsequent reaction of compound **13** and octylmagnesium bromide with Ni(dppp)Cl_2_ gave 3,3′-dioctyl-2,2′-bifuran (**15**) with a 74% yield. Stille coupling of dibromo derivative **16** (obtained from the bromination of **15** using NBS) with [2,2′:5′,2′′-terfuran]-5-yltributylstannane (**11**) yielded dioctyl-substituted octafuran **5**.

Oligomer **2** has the highest octyl chain to furan ratio (1 : 3), while oligomer **4** has the lowest octyl chain to furan ratio (1 : 7). In order to synthesize polymers having at least a 1 : 1 ratio of alkyl chain to furan ring, bromo groups present in compound **14** were replaced with alkyl chains. Kumada coupling of **14** with methylmagnesium bromide gave compound **6** (Scheme 3) with a moderate yield of 51%. The tetramethyl quaterfuran **7** (Scheme 3) was synthesized by lithiation of the monobromo precursor **18** followed by coupling with CuCl_2_.

### Electrochemical polymerization and characterization

Electropolymerization of furan monomer could be achieved only at high potentials (*ca.* 1.8 V *vs.* SCE),^5,12a^ at which point ring opening and irreversible degradation leads to non-conjugated and low quality polyfurans. Thus, the electropolymerization potential needs to be lowered. This is achieved by using oligofurans rather than furan monomer as the repeat units.^3b,11b,23^ Starting from terfuran, the oxidation potential of oligofurans drastically decreases, and the polymerization process proceeds below 1.0 V *vs.* SCE.^11*b*^ Such low oxidation potentials allowed the preparation of polyfurans reported herein under air at ambient conditions, which can be beneficial for their possible applications.

**Fig. 1:**
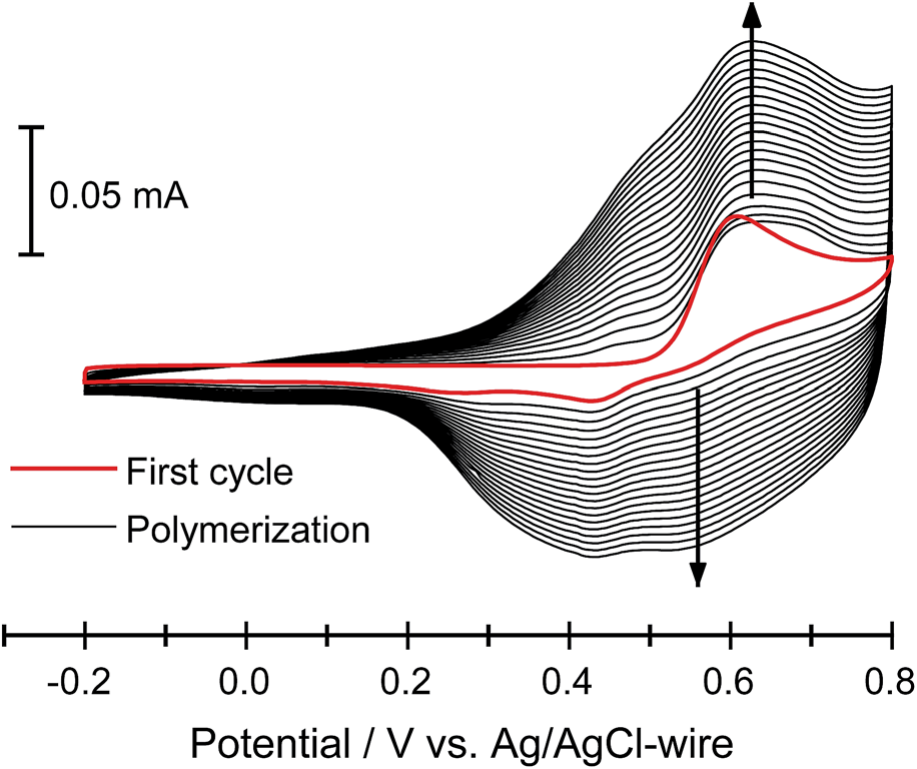
Electrochemical deposition of **3** (0.1 mM) in 0.1 M TBACF_3_SO_3_/ACN at 50 mV s^–1^ on an ITO-coated glass slide.

The electrodeposition of polymers **P1–P7** has been performed both potentiodynamically and potentiostatically. The best polyfuran films were obtained using a 0.1–1 mM concentration of the oligomer in an ACN solution containing 0.1 M TBACF_3_SO_3_ as the electrolyte. In general, for potentiodynamic polymerization, the potential was scanned at 50 mV s^–1^ from –0.2 V to 0.8 V *vs.* Ag/AgCl-wire and for potentiostatic polymerization, a potential of 0.75 V *vs.* Ag/AgCl-wire was applied. Further optimization of polymerization conditions is possible for each of the oligomers (see Table S2, ESI‡). Smooth electrodeposition and the growth of insoluble, highly adhesive continuous polymeric films were observed on different types of working electrodes (*i.e.*, ITO-coated glass, Pt-disk, Au-coated quartz crystal and IDA). It is worth noting that the electrodes were not modified and specially cleaned as they were in the preparation of smooth polythiophenes.^24^ Representative cyclic voltammograms (CV) for the polymerization of **P3** (PFu_5_-C_8_) on ITO electrode, showing stepwise increases in the current over the course of polymerization, are displayed in Fig. 1 (for other polymers see Fig. S3, ESI‡).

Oxidation peak potentials are poorly resolved for oligomers **1**, **2**, **4** and **5** (Fig. S3‡). Therefore, the onset potentials for oligomer oxidation (*E*_ox_), measured from the first CV curve of the polymerization, were used for comparison between different oligomers (Table 1). As expected, both the introduction of an alkyl group into an oligomer and the elongation of the oligomer decrease the oxidation potential. For example, the *E*_ox_ for **P1** (PFu_3_) is 0.62 V, whereas for **P2** (PFu_3_-C_8_) and **P3** (PFu_5_-C_8_), it is 0.51 V and 0.43 V, respectively. Furthermore, polyfurans are obtained at lower oxidation potentials than polythiophenes, as shown by the oxidation onset potential of terfuran, which is 0.33 V lower than that of terthiophene.

**Table 1:** Onset potentials for **1–7** and T3 oligomer oxidation (*E*_ox_)

Oligomer	*E*_ox_,^*a*^ V *vs.* Ag/AgCl-wire
Fu_3_ (**1**)	0.62
Fu_3_-C_8_ (**2**)	0.51
Fu_5_-C_8_ (**3**)	0.43
Fu_7_-C_8_ (**4**)	0.40
Fu_8_-2C_8_ (**5**)	0.37
Fu_3_-3C_1_ (**6**)	0.44
Fu_4_-4C_1_ (**7**)	0.41
T_3_	0.95

^*a*^For approximate oxidation peak potentials see Table S1.

It is well known that the electrolyte, solvent, oligomer concentration, and method of electropolymerization drastically affect the quality of the resulting polymeric films.^1d,23,25^ We found that TBACF_3_SO_3_/ACN electrolyte (see Fig. S4‡ for others) and concentrations of monomers lower than usually used^3b,11b^ produced better polyfurans. Electrolyte anions and solvent could affect the stability of intermediate σ-dimers, while lower concentrations could reduce π-dimerization of cation radicals that are produced during electropolymerization.^1*d*^

**Fig. 2:**
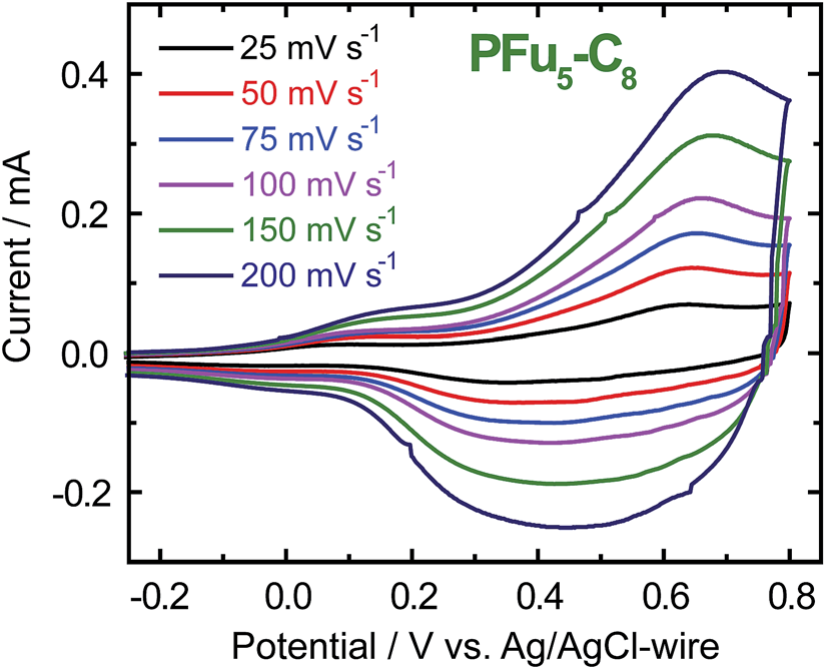
CV of **P3** film on an ITO coated glass electrode in monomer-free 0.1 M TBACF_3_SO_3_/ACN electrolyte at different scanning rates.

CVs of polyfurans in monomer-free TBACF_3_SO_3_/ACN electrolyte showing broad oxidation and reduction peaks (Fig. 2 and S5‡), are similar to those of other conducting polymers.^1d,3b^ It is difficult to determine accurate oxidation potentials for polyfurans from the obtained CVs. Nevertheless, oxidation potentials could be estimated from *in situ* conductivity measurements (see below).

### Optical and structural properties

**Fig. 3:**
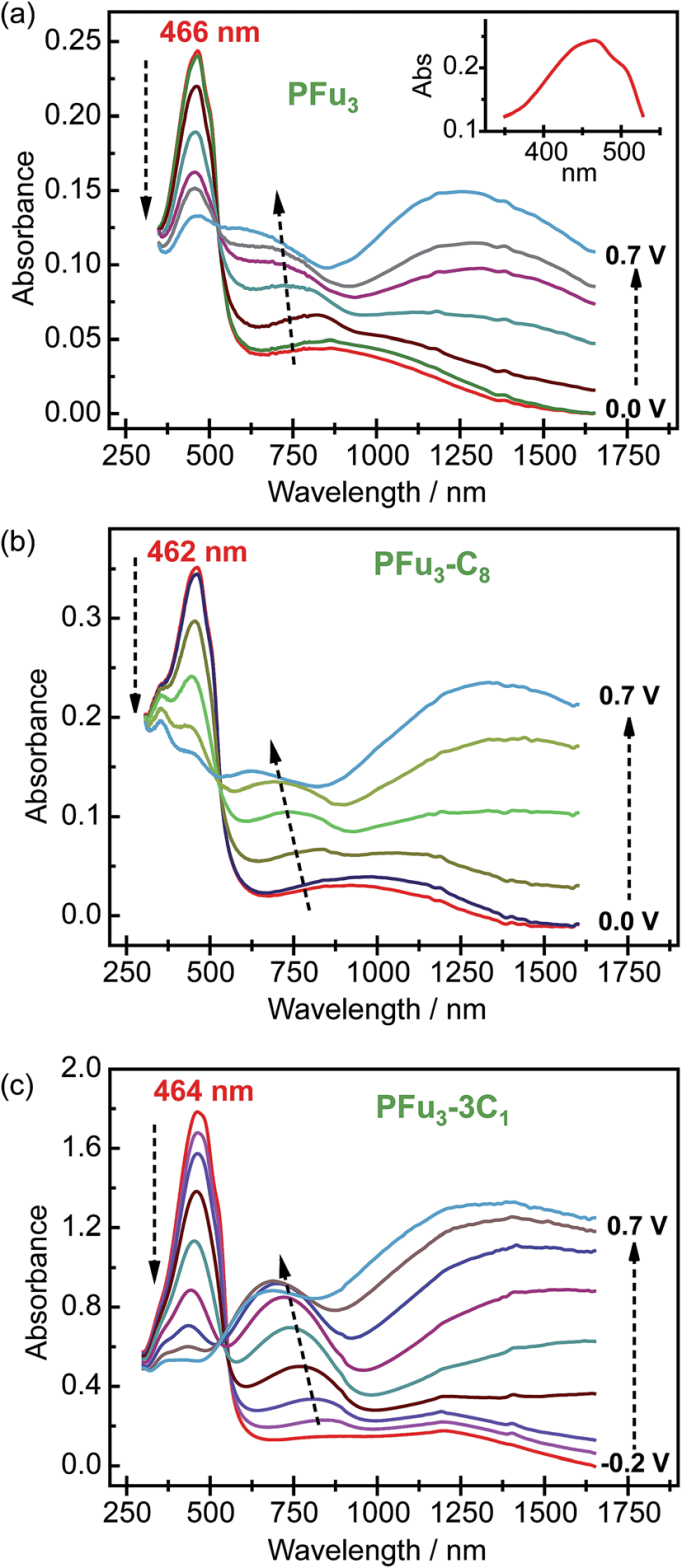
Spectroelectrochemistry of (a) **P1**, (b) **P2**, and (c) **P6** on an ITO coated glass electrode in monomer-free 0.1 M TBACF_3_SO_3_/ACN electrolyte. Inset (a) shows closer look on the vibronic shoulders of the main absorption peak of **P1** at 0.0 V. Stated potentials are *vs.* Ag/AgCl-wire.

Spectroelectrochemical studies of the electrochemically produced **P1–P7** films on ITO electrodes were performed to evaluate their electronic structure. All prepared polymers exhibited well-defined spectroelectrochemical responses, namely, a reversible and distinct change in the absorption spectrum during the redox processes (doping/dedoping). Fig. 3 shows representative spectroelectrochemical spectra for **P1**, **P2**, and **P6** films (for other polymers see Fig. S8‡). During polymer oxidation, the absorption peak of the neutral polymer film at around 460 nm decreases and two peaks at around 750 nm and 1250 nm develop. In addition, the isosbestic point is observed at *ca.* 530 nm for **P1–P5** and at *ca.* 550 nm for **P6** and **P7**. Importantly, the presence of one isosbestic point indicates interconversion between neutral and cation-radical (polaron) states.^26^ Only the 0.7 V spectral trace of **P6** (Fig. 3c) shows beginning of bipolaron formation, which is consistent with the decrease in electrical conductivity (see below).

The measured *λ*_max_ values of neutral polyfurans are in the range of 456–466 nm (Table 2). This range is red-shifted compared with most of previously reported values of <420 nm.^10c,11a,c,d,12b^ This suggests that much longer effective conjugation lengths are obtained in the current work. We note that Kanatzidis *et al.* reported^11*b*^ an absorbtion maximum of 468 nm for PFu_3_. However, their spectrum lacks vibronic shoulders, which are observed in all our spectra. We estimate the effective conjugation length in prepared polyfurans to be more than 25 furan units, as can be deduced from extrapolation of the *λ*_max_ values of α-oligofurans (see Fig. S25‡).

**Table 2:** Experimental and calculated electronic and optical properties for neutral **P1–P7**

Polymer	*λ*_max_, nm	Optical band gap^*a*^, eV	*E*_g_ (calc)^*b*^, eV	HOCO (calc)^*b*^, eV	LUCO (calc)^*b*^, eV
PFu_3_ (**P1**)	466	2.31	2.41	–4.36	–1.95
PFu_3_-C_8_ (**P2**)	462	2.31	2.35	–4.20	–1.85
PFu_5_-C_8_ (**P3**)	463	2.32	2.37	–4.23	–1.88
PFu_7_-C_8_ (**P4**)	458	2.29	2.38	–4.29	–1.90
PFu_8_-2C_8_ (**P5**)	460	2.31	2.36	–4.23	–1.88
PFu_3_-3C_1_ (**P6**)	464	2.22	2.25	–4.02	–1.78
PFu_4_-4C_1_ (**P7**)	456	2.22	2.26	–4.03	–1.77
PT_3_ ^*c*^	475	2.02	2.05	–4.61	–2.56

^*a*^The optical band gaps were obtained from the extrapolation of the linear part of the (*αhν*)^2^ *vs. hν* plot to (*αhν*)^2^ = 0, where *α* is the absorption coefficient.

^*b*^Calculated using the PBC/B3LYP/6-31G(d) level.

^*c*^For comparison, PT_3_ was prepared and measured in this work at same conditions as polyfurans.

The optical band gaps of polyfurans estimated from the absorption spectra (Table 2) for unsubstituted **P1** and partially alkyl substituted **P2–P5** are *ca.* 2.3 eV. By contrast, for polyfurans **P6** and **P7** substituted with one methyl group per furan ring, the band gaps are decreased to *ca.* 2.2 eV. This observation is consistent with DFT calculations, which showed that alkyl substitution reduces band gaps (Table 2 and S3‡). Moreover, calculations (Table 2) showed that this reduction in band gap occurs because of the increase in the highest occupied crystal orbital (HOCO) energy (*i.e.* the energy of the valence band), which is partially offset by an increase in the lowest occupied crystal orbital (LUCO) energy (*i.e.* the energy of the conduction band). Consequently, the oxidation potential of alkyl-substituted polyfurans decreased. The increase of the HOCO energy is consistent with the electron donating property of alkyl groups.

**Fig. 4:**
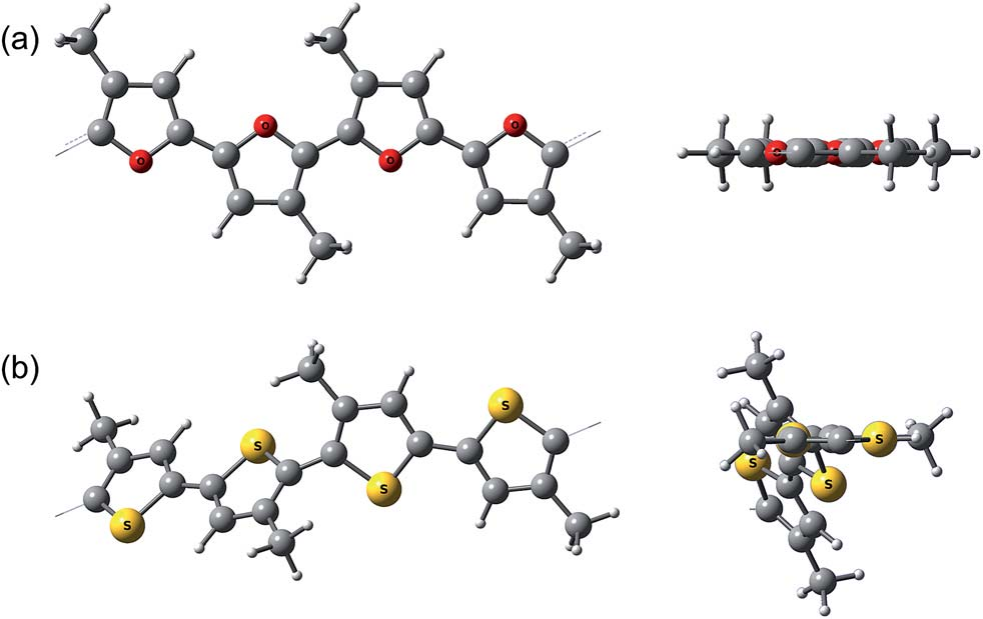
Calculated (PBC/B3LYP/6-31G(d)) geometry of (a) **P7** and (b) polythiophene analog of **P7**, PT_4_-4C_1_. A side view and a view along the polymer backbone.

Interestingly, the peak of neutral polyfuran films shows red-shifted shoulders (Fig. 3 and S8‡), probably because of the vibronic coupling.^27^ The presence of vibronic coupling may indicate that the polymer backbone is highly rigid, planar and effectively π-conjugated. We have shown that oligofurans exhibit high rigidity,^14,28^ as evident from calculated twisting potentials which are higher than for oligothiophenes. Similar computational results have been recently shown for polyfurans.^29^ Consequently, the π-conjugation and planarity of polyfurans should not be distorted by various substitution of the polyfuran backbone as strongly as it is in the polythiophene family. Indeed, DFT geometry optimization (PBC/B3LYP/6-31G(d)) of polyfurans **P1–P7** shows that the polyfuran backbone remains planar upon alkyl substitution (Fig. 4a and S10‡). In contrast, in polythiophene PT_4_-4C_1_, which is a thiophene analogue of **P7**, the polymer backbone deviates strongly from planarity (Fig. 4b), distorting the π-conjugation. As a consequence, the calculated band gap for PT_4_-4C_1_ increases to 2.9 eV from 2.0 eV for planar unsubstituted polythiophene.^29,30^ Whereas measured optical and calculated band gaps for polyfurans **P1–P7** are not increased upon alkyl substitution.

### IR spectroscopy

**Fig. 5:**
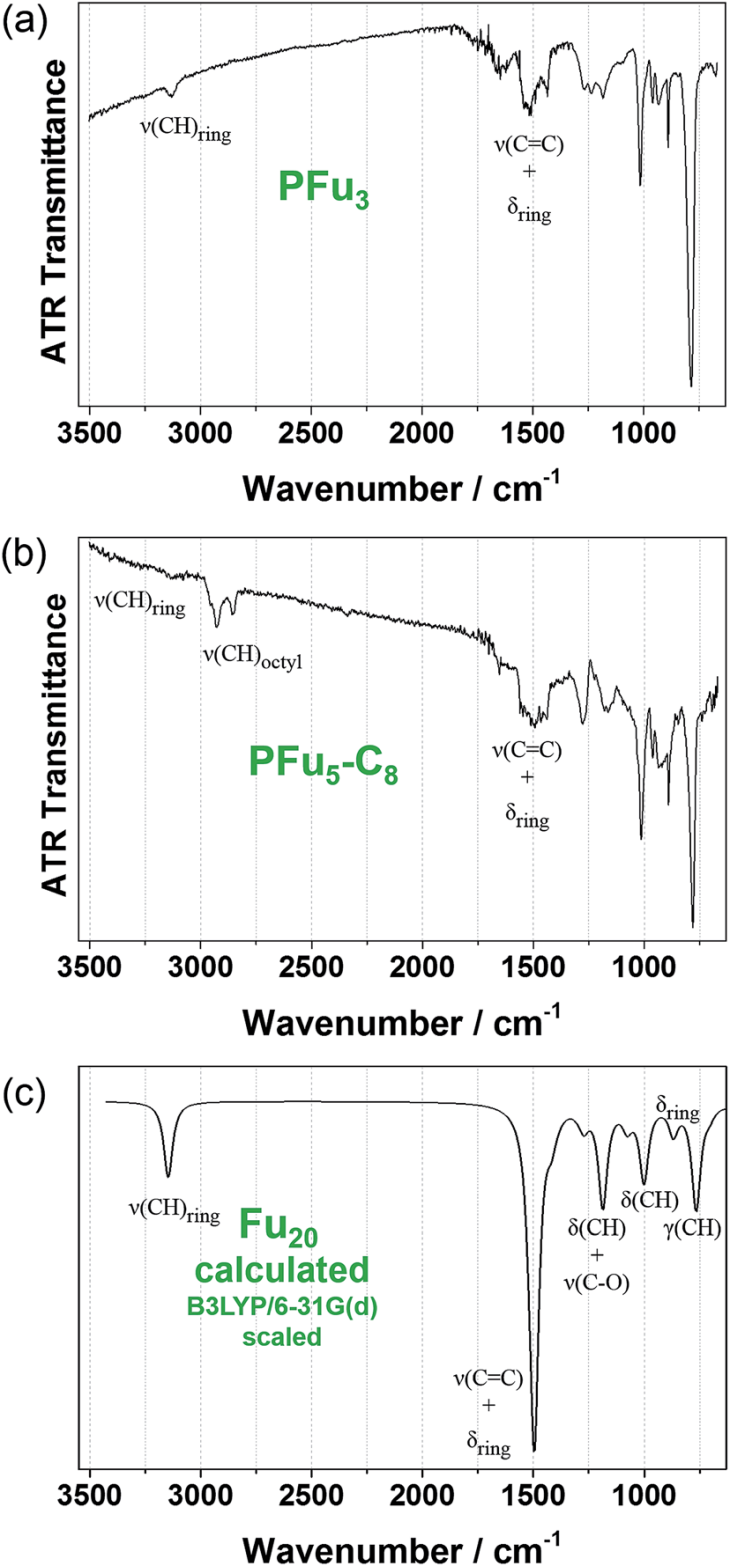
Experimental FTIR-ATR spectra: (a) of **P1** and (b) of **P3** films electrodeposited on platinum foil. (c) DFT B3LYP/6-31G(d) calculated IR spectrum of Fu_20_ (frequencies were scaled by 0.96 (ref. 31)).

Structural defects in the conjugated backbone of polyfuran can be revealed by IR spectroscopy.^32^ Previously reported polyfurans showed defects, such as ring-opening and β-coupling, which were observed by stretching vibrational modes of the aliphatic C–H bond (∼2900 cm^–1^), carbonyl C=O bond (∼1720 cm^–1^), and O–H bond (∼3500 cm^–1^).^10b,11a–c^ The measured ATR-FTIR spectra of **P1** and **P3** films on Pt-foil (Fig. 5a and b) show little structural defects for **P1** and almost none for **P3** and are in good agreement with the calculated spectrum of model Fu_20_ (Fig. 5c, Table S4 and Fig. S12‡) and with previous IR data.^10b,11c,33^ The IR spectra of **P1** and **P3** are similar, showing the same bands except for the aliphatic C–H stretching mode of the octyl substituents at around 2900 cm^–1^.

### *In situ* conductivity

The conductivity of conjugated polymers strongly depends on many factors, particularly the method of preparation, the presence of impurities, humidity, and the measurement technique.^1*b*^ Owing to their simplicity as well as their ability to obtain a potentiodynamic response, *in situ* conductivity measurements using an interdigitated array (IDA) microelectrode^34^ or two-band electrode^35^ have found wide use.^36^ However, obtaining the absolute value of polymer film conductivity is not straightforward because it depends on many factors that are hard to define, such as non-trivial geometrical factor of the electrode, non-linear dependence of the conductance on film thickness, and undefined contact resistances. Thus, we report conductance values in comparison to that of poly(terthiophene) (PT_3_), which was electrochemically prepared and measured under the same setup. Additionally, we compare the conductance values of our polyfurans to those of drop-cast commercial poly(3,4-ethylenedioxythiophene) poly(styrenesulfonate) (PEDOT:PSS) film on the same type of electrode. The conductivity of electrochemically prepared PT_3_ film^7*a*^ and that obtained from commercial PEDOT:PSS^37^ is estimated to be on the order of 1 S cm^–1^.

**Fig. 6:**
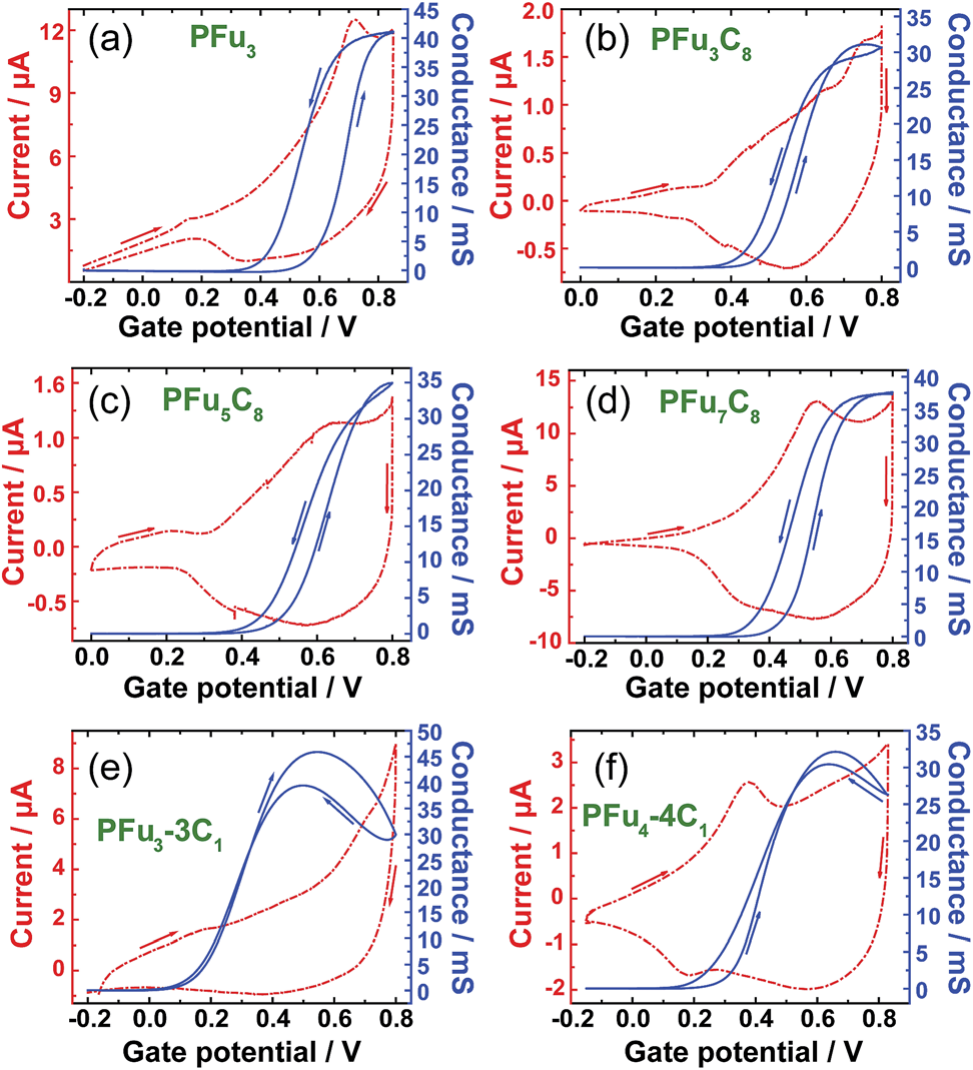
Cyclic voltammograms (dashed line) and *in situ* conductance measurements (solid line) for: (a) **P1**, (b) **P2**, (c) **P3**, (d) **P4**, (e) **P6** and (f) **P7** in monomer-free TBACF_3_SO_3_/ACN using a 10 μm interdigitated array gold microelectrode. Sweep rates are 10 mV s^–1^ for cyclic voltammograms with a 10 mV probe potential for conductivity measurements. Stated potentials are *vs.* Ag/AgCl-wire.

Fig. 6 shows simultaneous measurements of CV and conductance of **P1–P4**, **P6** and **P7** films. All obtained polyfurans show maximum conductances comparable to those of PT_3_ and PEDOT:PSS films (Table 3, Fig. S15‡). Thus electrical conductivities of doped polyfurans are in the order of 1 S cm^–1^. This conductivity is much higher than most published values, which range from 10^–2^ to 10^–5^ S cm^–1^.^5^

**Table 3:** Conductance and half-maximal conductance potential (*E*_hc_) for forward scan in **P1–P4**, **P6**, **P7**, PT_3_ and PEDOT

Polymer	Conductance (max), mS	*E*_hc_ ^*a*^, V
PFu_3_ (**P1**)	43	0.69
PFu_3_-C_8_ (**P2**)	32	0.58
PFu_5_-C_8_ (**P3**)	38	0.62
PFu_7_-C_8_ (**P4**)	37	0.55
PFu_3_-3C_1_ (**P6**)	46	0.30
PFu_4_-4C_1_ (**P7**)	32	0.44
PT_3_	42	0.87
PEDOT	>72^*b*^	–0.21
PEDOT:PSS^*c*^	50	—

^*a*^Half-maximal conductance potential (*vs.* Ag/AgCl-wire) for the forward scan.

^*b*^Limited by the resistance of the IDA electrode contacts (Fig. S15).

^*c*^Drop-cast film.

Each polymer undergoes transition to conductive state at different potentials (Fig. 6 and S15‡). We used the potential at half-maximal conductance (for the forward scan) (*E*_hc_) for comparison between polymers. As evident from Table 3, *E*_hc_ depends on the degree of alkyl substitution, *i.e.*, *E*_hc_ decreases with increasing degree of alkyl substitution (Table 3). For example, the value of *E*_hc_ for fully methylated polyfuran **P6** is 0.39 V is lower than that of the unsubstituted **P1**. Since, the transition to conductive state is associated with the oxidation of a conjugated polymer, we can conclude that the oxidation potential of polyfurans is also decreased by alkyl substitution. This is in the agreement with DFT calculations, which show increase in energy of the HOCO as the degree of alkyl substitution increases (Table 2 and S3‡). Furthermore, for polyfurans, the *E*_hc_ potentials are lower than that of PT_3_ but higher than that of PEDOT, which is consistent with the higher oxidation potential of PT_3_ (ref. 38) and the lower oxidation potential of PEDOT.^39^

We observe that the conductivity of fully methylated polyfurans **P6** and **P7** reaches maximum at 0.5 V and 0.6 V, respectively, and decreases at higher potentials. This observation may suggest that the charge carriers are polarons. Upon further electrochemical oxidation, their conductivity decreases (reversibly) as bipolarons are formed. However, this behavior is not observed in polyfurans **P1–P4** due to degradation of the polyfuran films at potentials above 0.8 V, where their conductivity decreases irreversibly.

### Electrochemical stability

The electrochemical stability of conducting polymers is of utmost importance for their use in electrochemical systems, such as batteries, electrocatalytic materials, sensor devices, and other electronic components.^40^ Electrochemical degradation of conductive polymers, such as polypyrrole, polythiophene, and polyaniline, has been studied extensively.^40^ However, very little has been reported on the electrochemical degradation of polyfuran.^10b,11c^

#### (a) Redox current stability

**Fig. 7:**
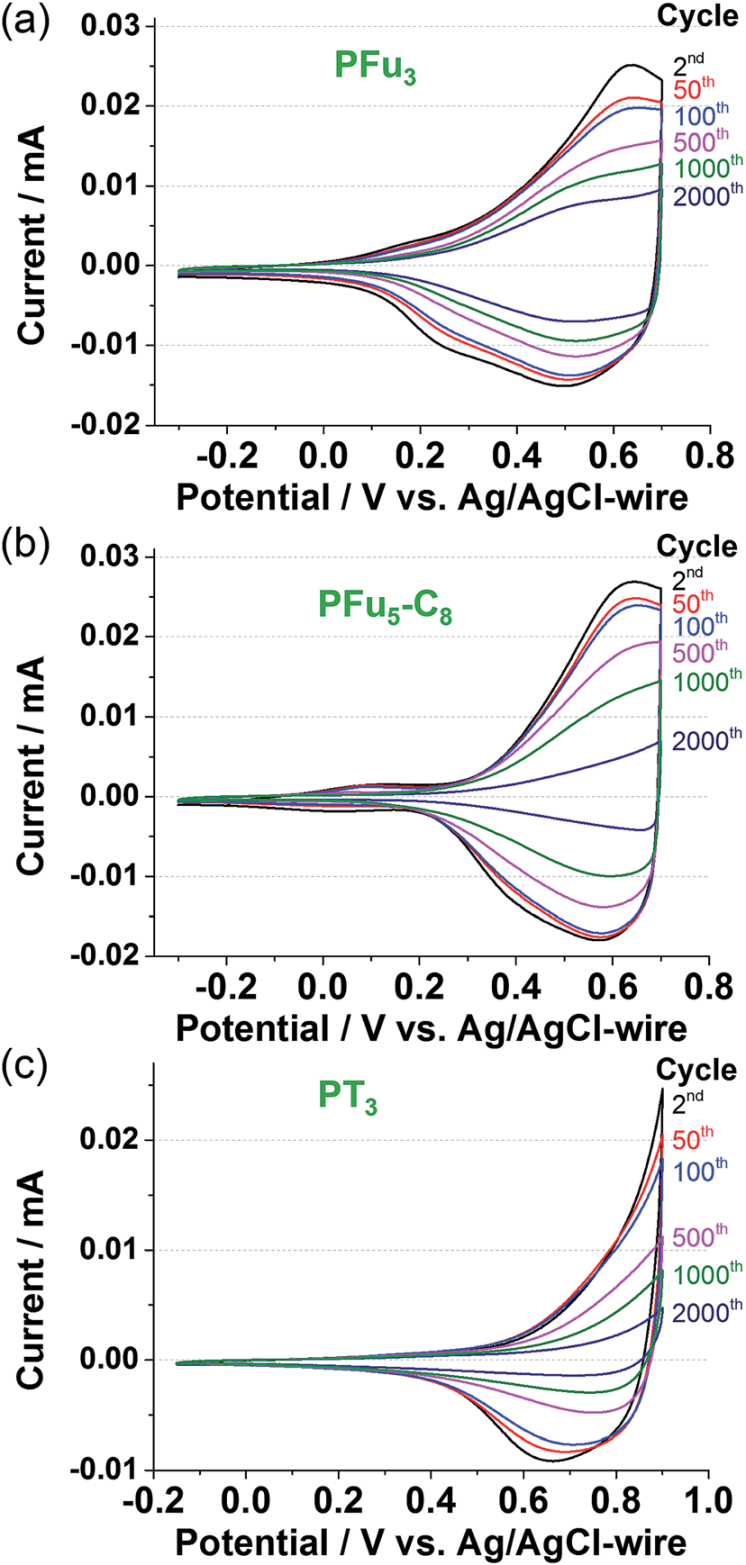
Long term CV at 100 mV s^–1^ of polymer films: (a) **P1**, (b) **P3**, and (c) PT_3_ on Pt-disk electrode in monomer-free 0.1 M TBACF_3_SO_3_/ACN electrolyte.

The electrochemical cyclability of the polyfurans was tested using cyclic voltammetry between –0.3 to 0.7 V *vs.* Ag/AgCl-wire in a monomer-free TBACF_3_SO_3_/ACN electrolyte solution for up to 2000 cycles in the air under ambient conditions (Fig. 7). Polyfurans **P1** and **P3** show reasonable electrochemical stability, with oxidation current remaining at *ca.* 50% after 1000 cycles. This is considerably better than the values reported for polyfurans retention of redox activity (40–50% over 100 cycles).^10b,11c^ In order to properly compare the stability of polyfurans with that of PT_3_, the potential scan window was shifted by 0.2 V towards higher potentials. The reasoning for this stems from the fact that PT_3_ has a higher oxidation potential.^3b,41^ Under these conditions, polyfurans showed slightly better stability than PT_3_ (Fig. 7c), because PT_3_ undergoes faster degradation due to higher applied potential that is required to induce its conductivity. Furthermore, as shown in Fig. S13,‡ the addition of 1% v/v water to the electrolyte solution had a small effect on the electrochemical stability of polyfuran **P3**. As expected, the electrochemical stability upon cycling over 0.0–0.5 V was much higher (Fig. S14‡). **P1** and **P3** show only a 25% reduction in oxidation current after 10 000 cycles (Fig. S14a and c‡).

#### (b) Optical switching and stability

**Fig. 8:**
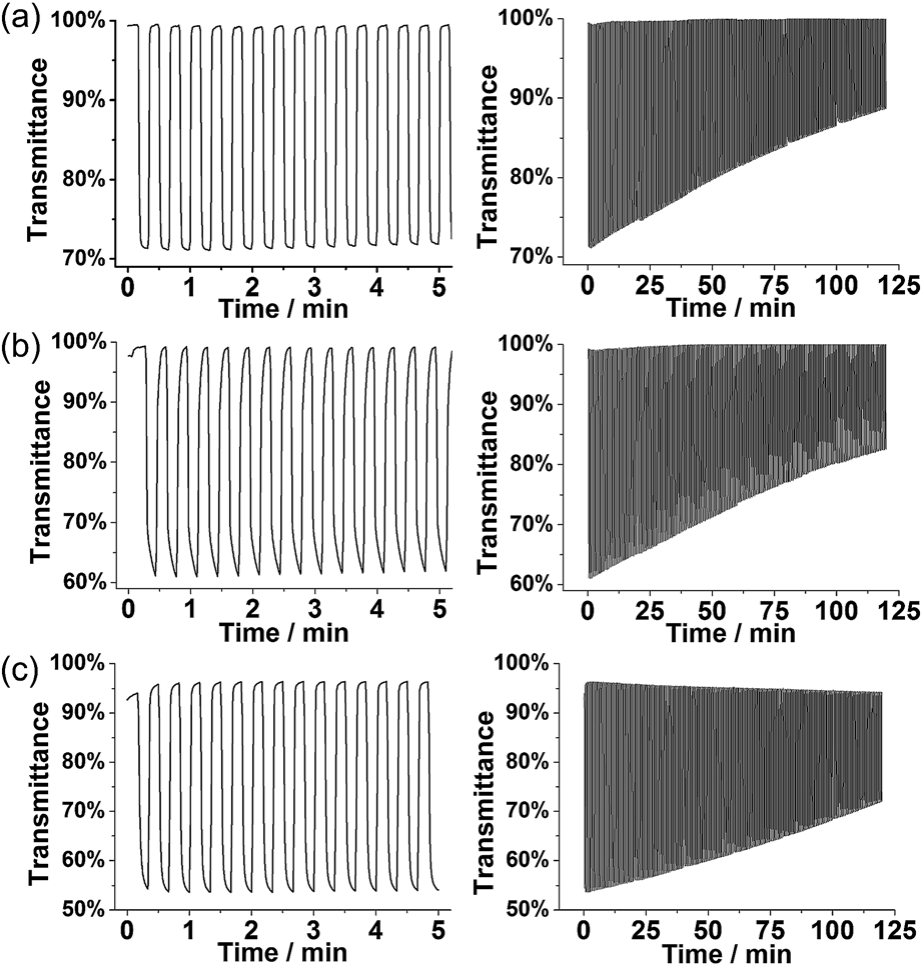
Optical switching and stability of polymer films: (a) **P1** at 1250 nm, (b) **P3** at 1350 nm, and (c) PT_3_ at 1275 nm on ITO coated glass electrodes in monomer-free 0.1 M TBACF_3_SO_3_/ACN electrolyte. Voltage pulses of 0.0 V and 0.7 V (for **P1** and **P3**) and of 0.0 V and 0.9 V (for PT_3_) were applied for 10 s.

Optical switching was measured for doped **P1**, **P3**, and PT_3_ at the maximum absorption peak wavelength in the NIR region (see spectroelectrochemistry above), within which the degraded and non-electroactive polyfurans show no absorbance. The stability of optical switching was evaluated by applying repetitive potential pulses (10 s length) of 0.0 V and 0.7 V for polyfurans, and 0.0 V and 0.9 V for PT_3_ for prolonged time (2 h) (Fig. 8). For complete doping and achievement of maximal NIR absorbance, higher potential was needed for PT_3_.

The degraded optical activities (*i.e.*, difference in transmittance of undoped and doped state) obtained after 120 min of cycling (for a total of 360 cycles) were found to be 39%, 45%, and 51% for **P1**, **P3** and PT_3_ of the starting activity, respectively. Thus, polyfurans **P1** and **P3** showed only slightly lower stabilities compared with PT_3_, but are much better than that reported for a PFu/PEDOT device.^10*c*^

#### (c) Stability in conductive state

**Fig. 9:**
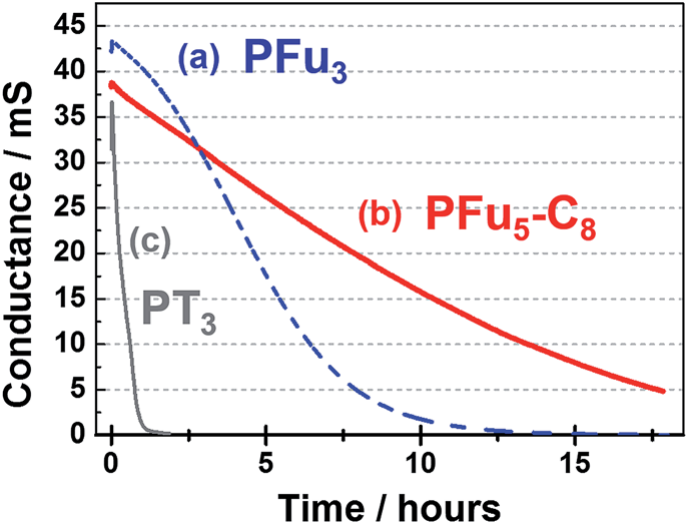
Stability of the conductive state of polymer films: (a) **P1** at 0.7 V, (b) **P3** at 0.7 V, and (c) PT_3_ at 0.9 V on an IDA electrode in monomer-free 0.1 M TBACF_3_SO_3_/ACN electrolyte.

The stability of the conductive state of the **P1**, **P3** and PT_3_, was tested on an IDA electrode by applying a constant potential for a prolonged time (up to 18 h) in air under ambient conditions (Fig. 9). A potential of 0.7 V was used for polyfurans **P1** and **P3**. For polythiophene PT_3_, a potential of 0.9 V was used. These potentials correspond to slightly lower potential at which the polymers exhibit maximum conductance (Fig. 6).

As shown in Fig. 9, polyfurans **P1** and **P3** show much higher stabilities toward continuous potential application than PT_3_, whose lower stability is likely due to the high potential (0.9 V) needed for oxidation (doping), which in turn, is expected to break the conjugation of the backbone *via* side reactions.^42^ Thus, the lower potential required for doping the polyfurans is advantageous because it minimizes the occurrence of backbone degradation under ambient conditions.

### Morphology

Polymer morphology has a significant effect on polymer charge–transport properties.^1*b*^ Controlled film thickness, surface smoothness, and uniformity are important considerations for application of conductive polymers in thin film devices (where high surface area is not required, such as sensing), including photovoltaic cells and OFETs. Electrochemical deposition has some advantages over solution processing of conjugated polymers. It combines polymer formation and deposition in one-step and bypasses the need for polymer isolation and purification. As such, it has found use in fabrication of organic electronic devices.^43^ However, it is difficult to obtain smooth and homogeneous thin films by electropolymerization, necessitating the optimization of electrodeposition.^1*d*^ For example, the root mean square roughness (*r*_rms_) of PT_3_ on ITO is 173 nm (Fig. S18‡) and PT on ITO is 25–174 nm.^24^ To the best of our knowledge, the lowest *r*_rms_ value obtained for electrodeposited PEDOT films in organic media is 46 nm.^25*c*^ Smooth polythiophene films were obtained after modification of the working electrode,^24,44^ while smooth electrodeposited PEDOT films were obtained from microemulsions in aqueous solution.^45^ The polymer precursor approach (*i.e.*, use of inert polymer with pendant electroactive monomer units) was proposed to obtain thin films of less than 100 nm with controllable thickness.^46^ The drawback of this method, however, is that prior polymer synthesis is necessary.

**Fig. 10:**
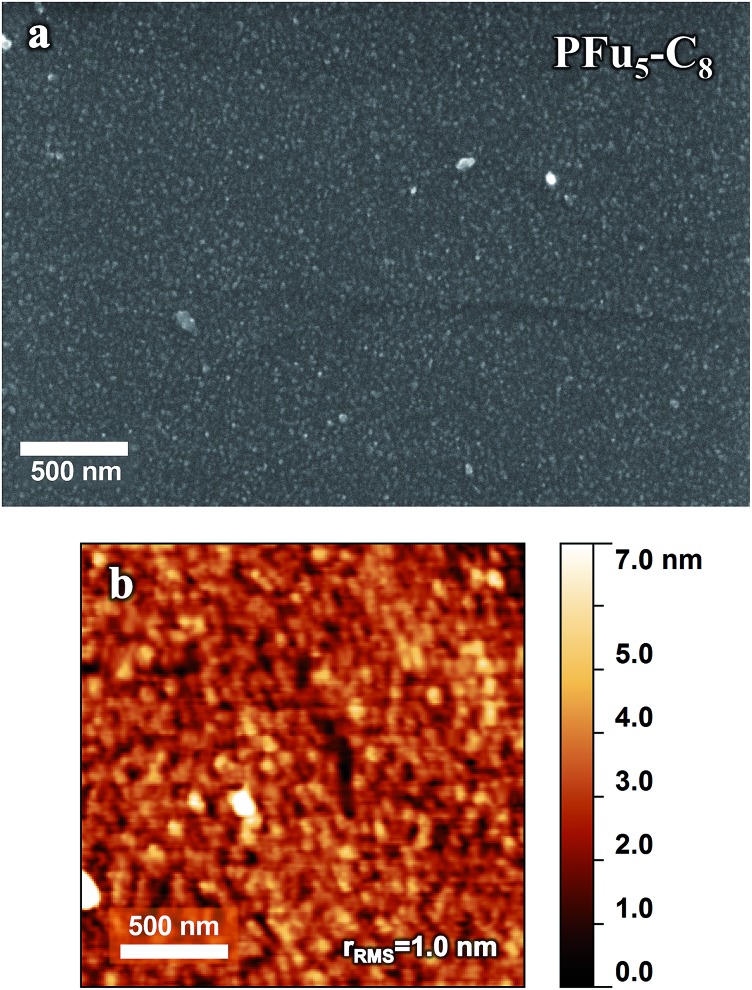
(a) SEM and (b) AFM images of **P3** film prepared on a HOPG electrode by CV polymerization using potential scanning from –0.2 V to 0.78 V for 1 cycle of 0.2 mM of monomer **3** in 0.1 M TBACF_3_SO_3_/ACN electrolyte. Film thickness is 10 nm.

As can be seen in Fig. 10 showing SEM and AFM images of representative example of **P3** on HOPG electrode (for the additional images of polyfurans on different electrodes see ESI‡), the prepared polyfuran films show excellent surface smoothness and complete coverage of all working electrodes used. In contrast, polythiophene exhibits separate islands of film and areas of bare electrode surface (see Fig. S17‡ for similarly prepared PT_3_).^47^ Additionally, polyfuran films have high adhesion to the electrode surface and are not easily detached. For example, they cannot be pulled off by sticking adhesive tape to the electrode, as in case of poly(3-methylthiophene) films.^48^ Additionally, films were found to be completely insoluble in organic solvents, such as THF and toluene.

**Fig. 11:**
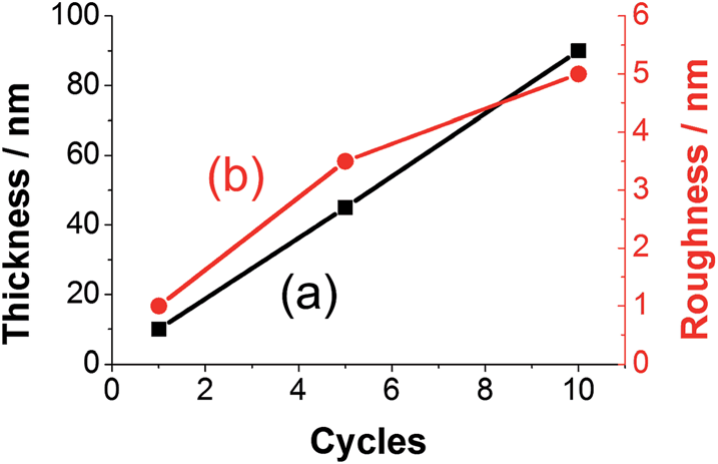
Dependence of (a) thickness and (b) roughness on number of CV cycles used for polymerization of **P3** on HOPG electrode.

The thickness of prepared polyfuran films can be easily controlled and tailored for the specific application. For instance, the film thickness of **P3** linearly depends on the number of CV cycles used in the polymerization (Fig. 11). The thickness of 10 nm on HOPG electrode was obtained after 1 cycle (Fig. 10), 45 nm after 5 cycles (Fig. S19‡) and 90 nm after 10 cycles (Fig. S20‡). The films show full coverage of electrodes with very smooth and homogeneous morphology. Although the *r*_rms_ value for the 10 nm thick film was only 1 nm, it increased with film growth, as evidenced by the *r*_rms_ values of 3.5 nm and 5 nm for 45 and 90 nm thick films, respectively (Fig. S20‡). For polyfuran films with thickness above 50 nm, formation of globular structures on the top of smooth film is observed on both HOPG and ITO electrodes (see Fig. S20–S22‡). Polyfuran film roughness on an ITO electrode (Fig. S21–S22‡) is higher than on HOPG and is due to inherent higher roughness of the electrode (*i.e.*, *r*_rms_ = 4.6 nm for bare ITO, Fig. S23‡).

## Discussion

Overall, we have shown that by careful selection of starting oligomers and electropolymerization conditions (*i.e.*, electrochemical method, monomer concentration, electrolyte and solvent), polyfurans of good quality are obtained. By lowering the polymerization potential using long oligofurans as the starting monomers, the polyfurans could be prepared in open air under ambient conditions.

The measured high conductivity on the order of 1 S cm^–1^ for doped polyfuran films may be attributed to better quality of the prepared polyfurans when compared with previous reports.^5^ This increased quality is manifested in the longer effective conjugation lengths (estimated to be above 25 furan units based on oligomer data, Fig. S25‡) revealed by absorption spectra of the reduced (dedoped) polyfuran films, which show considerable red-shifted (456–466 nm) absorption maxima compared to most reported polyfurans.^11a,c,d,12b^ Additionally, IR spectroscopy showed almost no structural defects, including ring opening and β-coupling in the backbone. Moreover, absorption spectra of the neutral polyfurans show vibronic coupling, indicative of well-ordered, planar, and rigid polymers. Although similar vibronic coupling is also observed in highly conductive samples of PT^49^ and PEDOT,^27*b*^ they were not previously observed in electrochemically prepared polyfurans. Furthermore, the measured conductivity of polyfurans is comparable to the conductivity of electrochemically prepared poly(oligothiophene)s.^7*a*^ Thus, we are convinced that the previously reported low conductivities^5^ are due to preparation problems, and are not an intrinsic property of polyfurans.

We have studied the effect of alkyl substitution on the properties of polyfurans. DFT calculations predict reductions of the band gaps, as well as oxidation potentials of polyfurans, with increasing alkyl substitution ratio (see Table S3‡). Indeed, compared to **P1**, we experimentally observe a reduction of around 0.08 eV (Table 2) in optical band gaps in fully methylated polyfurans **P6** and **P7**. Although we cannot reliably measure the oxidation potentials from cyclic voltammetry, we do qualitatively observe that the onset of the broad oxidation peak is shifted to lower potentials for **P6** and **P7**. Another estimation of oxidation potentials can be obtained from the potential at half-maximum conductance *E*_hc_, which is significantly decreased upon alkyl substitution (*e.g.*, *E*_hc_ = 0.69 V *vs.* Ag/AgCl-wire for PFu_3_ (**P1**) and *E*_hc_ = 0.30 V for PFu_3_-3C_1_ (**P6**)). Furthermore, *E*_hc_ for polyfurans is lower than that of PT_3_ (*E*_hc_ = 0.87 V). Therefore, when compared to polythiophenes, a lower oxidation potential of polyfurans serves as an advantage. The lower doping potential minimizes the occurrence of the destructive side reactions (*e.g.*, with water and oxygen). Moreover, oligofurans show lower oxidation potential compared to oligothiophenes, as evidenced by the onset potentials for oligomer oxidation (0.62 V for terfuran and 0.95 V for terthiophene).

As shown by DFT calculations^29^ and absorption spectra (Fig. 4 and S11‡), polyfurans have the further advantage of being more rigid than polythiophenes. High rigidity of polyfurans is also consistent with the experimental observation that regioirregular methylated polyfurans **P6** and **P7** show lower band gaps and oxidation potentials. This is advantageous for future development of furan-based polymers, where regioregular arrangement of different functional groups (otherwise needed in the case of polythiophenes to keep the efficient π-conjugation, *e.g.*, rrP3HT)^50^ is no longer mandatory.

We have carefully studied the stability of electrochemical, optical, and conductive properties of polyfurans in open air under ambient conditions. The stability of the redox activity and conductivity over time showed that polyfurans are actually more stable than polythiophene PT_3_ under similar conditions. This stability is attributed to lower potential needed for doping of polyfurans. The optical switching stability of polyfurans is found to be comparable with that of PT_3_, and it is much better than the previously reported stability of the optical switching of a PFu/PEDOT device at 415 nm. Previous studies showing optical switching degradation after only 90 s then is likely attributable to the polyfuran's poor quality, as suggested by the low absorption maximum (*λ*_max_ = 420).^10*c*^ Therefore, we believe that the earlier reported instabilities of polyfurans are also due to problems in their preparation.

We have identified two phases of the polyfuran film growth. Initially (up to *ca.* 50 nm thickness), we observe a smooth thin film covering the whole electrode surface is formed. As the film continues to grow, globular bumps and aggregates are observed on top of the smooth film surface. As a result, the roughness of the surface increases. For instance, in **P3** on HOPG, the *r*_rms_ value was *ca.* 5 nm for the underlying film and *ca.* 17 nm for the full scanned area (Fig. S20‡). The formation of the structures on top of homogenous film is consistent with electrochemical polymerization where a new polymer forms at surface points with a higher conductivity.

It is worth noting that prepared polyfuran films have lower roughness than most other electrochemically-prepared films under similar conditions,^24,25c,44–46^ *i.e.*, in organic media electrolyte, in ambient environment, without electrode surface modifications and extensive electrode cleaning. This is certainly an advantage for technological applications of polyfurans.

## Conclusion

Using long oligofurans as precursors, we were able to obtain and thoroughly characterize polyfuran films with different alkyl substitutions that exhibit high stability, are spectroelectrochemically active, show good conductivity in the open air under ambient conditions, and have smooth morphology. These properties are considerably improved from any other known polyfuran prepared electrochemically. Additionally, we showed that polyfurans exhibit high rigidity, which allows many different substituents to be introduced without distorting the planarity and conjugation of the polyfuran backbone. We showed that regioirregular alkyl-substituted polyfurans do not lose their conjugation, and moreover exhibit reduced band gaps.

Polymerization of oligofurans using considerable lower potential than parent furan allowed us to obtain stable polyfurans with very good properties. This is in contrast to the previous belief that polyfurans are intrinsically unstable and possess low conductivity. We believe that this work will establish polyfurans as a fundamental series of conductive polymers with important properties, opening up future applications for polyfurans as conductive polymers.
