# Supplementary material for: Conducting polyfurans by electropolymerization of oligofurans
Source: Chem Sci. 2014 Oct 17;6(1):360–71. doi: 10.1039/c4sc02664k (PMC5586207; doi:10.1039/c4sc02664k)
Supplement: Supplementary file 1 [file SC-006-C4SC02664K-s001.pdf]

Electronic Supplementary Material (ESI) for Chemical Science.
This journal is © The Royal Society of Chemistry 2014

Conducting Polyfurans by Electropolymerization of Oligofurans

Dennis Sheberla, Snehangshu Patra, Yair H. Wijsboom, Sagar Sharma, Yana Sheynin, Abd-Elrazek Haj-Yahia, Adva Hayoun Barak, Ori Gidron and Michael Bendikov

Department of Organic Chemistry, Weizmann Institute of Science, Rehovot, Israel, 76100

Supplementary Information

Table of Contents

Supplementary Information 1
Experimental 2
Materials 2
Methods 6
In situ conductivity 7
Calculations 8
Additional Data 9
Electrochemical polymerization 9
Electrolyte Effect 12
CV of polymers 13
Spectroelectrochemistry 15
DFT calculations 16
Electrochemical Stability 20
Conductivity measurements 21
IR Spectroscopy 23
Morphology study 24
Estimation of polyfuran effective π-conjugation length from UV-Vis spectroscopy 30
EQCM study of polyfurans 30

Experimental

Materials

Compounds **1–7** and **10–18** were synthesized (see below; for the synthesis of compounds **1**, **10** and **11** see elsewhere[1]). All other compounds were used as is following their purchase from Sigma-Aldrich, with the exception of 3-bromofuran, which was purchased from CAPOT Chemical Company Limited. Heraeus Clevios™ P standard dispersion (PEDOT:PSS) was passed through 1.2μm syringe filter before use.

^{1}H and ^{13}C NMR spectra were recorded in solution on Bruker AVANCE III 300 MHz, 400 MHz and Bruker AV-500 MHz spectrometers using tetramethylsilane (TMS) as the external standard. Chemical shifts are expressed in ppm. High resolution mass spectra were measured on a Waters Micromass GCT Premier Mass Spectrometer using field desorption (FD) ionization. Differential scanning calorimetry (DSC) measurements were performed on a TA Q200 DSC instrument. UV-Vis-NIR absorption measurements were made on a JASCO V-570 spectrometer. Et_2O, THF and toluene were distilled from sodium/benzophenone under an atmosphere of dry argon prior to use. Columns were prepared with silica gel (60-230 mesh).

Synthesis of 3''-octyl-2,2':5',2'':5''-terfuran (2). $Pd(PPh_3)_4$ (0.024 g, 0.021 mmol) was added to a solution of 2,5-dibromo-3-octylfuran (0.10 g, 0.30 mmol) and 2-tributyltinfuran (0.21 g, 0.60 mmol) in dry toluene (5 mL), and the reaction mixture was refluxed under nitrogen overnight. The mixture was then cooled and evaporated. Purification of the residue by flash chromatography on silica gel (elution with hexane) afforded the title product **2** (0.05 g, 53% yield).^{1}H NMR (500 MHz, $CDCl_3$): δ 0.87 (t, *J* = 7 Hz, 3H), 1.23-1.64 (m, 12H), 2.68 (t, *J* = 7.5 Hz, 2H), 6.46 (dd, *J* = 3.3 Hz, 1.7 Hz, 1H), 6.48 (dd, *J* = 3.3 Hz, 1.7 Hz, 1H), 6.52 (s, 1H), 6.54 (d, *J* = 3.4 Hz, 1H), 6.60 (d, *J* = 3.3 Hz, 1H), 7.41 (br. s, 1 H), 7.46 (br. s, 1H) ppm. ^{13}C NMR (125 MHz, $CDCl_3$,): δ 14.3, 22.9, 25.2, 29.4, 29.5, 29.6, 30.1, 32.1, 105.4, 105.8, 108.8, 111.4, 111.7, 124.0, 141.1, 141.8, 142.0, 145.0, 146.6, 147.0 ppm. UV-Vis (CH_2Cl_2): λ_{max} = 330 nm. HRMS (FD): m/z calcd for $C_{20}H_{24}O_3$: 312.1725; found 312.1726.

Synthesis of 3''-octyl-2,2':5',2'':5'',2''':4''',2''''-quinquefuran (3). A solution of 2,5-dibromo-3-octylfuran (0.25 g, 0.74 mmol) in 10 mL toluene was added to a stirred solution of [2,2'-bifuran]-5-yltributylstannane (0.63 g, 1.49 mmol) in toluene (10 mL) containing $Pd(PPh_3)_4$ (0.17 g, 0.15 mmol) under an inert atmosphere of nitrogen. The solution was allowed to reflux overnight and then cooled to room temperature. The solvent was evaporated and the crude product was purified by column chromatography on silica gel with hexane as eluent to yield **3** as yellowish colored solid (0.13 g, 39 % yield). ^{1}H NMR (400 MHz, C_6D_6): δ 0.89 (t, *J* = 6.9 Hz, 3H), 1.17-1.43 (m, 10H), 1.55-1.65 (m, 2H), 2.71 (t, *J* = 7.7 Hz, 2H), 6.07-6.11 (m, 2H), 6.49-6.55 (m, 6H), 6.57 (s, 1H), 7.01-7.03 (m, 2H) ppm. ^{13}C NMR (100 MHz, C_6D_6): δ 14.3, 23.0, 25.5, 29.7, 29.8, 29.8, 30.2, 32.3, 105.6, 105.8, 107.3, 107.6, 107.8, 108.0, 109.8, 111.68, 111.71, 124.9, 141.6, 142.1, 142.2, 145.3, 146.1, 146.2, 146.4, 146.6, 146.8, 147.0 ppm. UV-Vis (CH_2Cl_2): λ_{max} = 390 nm. HRMS (FD): m/z calcd for $C_{28}H_{28}O_5$: 444.1937; found 444.1945.

Synthesis of 3''-octyl-2,2':5',2'':5'',2''':5''',2'''': 5'''',2''''': 5''''',2''''''-sepifuran (4). $Pd(PPh_3)_4$ (0.097 g, 0.084 mmol) was added to a solution of 2,5-dibromo-3-octylfuran (0.35 g, 1.03 mmol) and [2,2':5',2''-terfuran]-5-yltributylstannane (1.01 g, 2.04 mmol) in dry toluene (25 mL) and the reaction mixture was refluxed under nitrogen overnight. The mixture was then cooled and evaporated. Purification of the residue by flash chromatography on silica gel (elution with hexane : ethyl acetate 10:0.25) afforded the title product **4** (0.15 g, 26% yield).^{1}H NMR (500 MHz, C_6D_6): δ 0.88 (t, J = 7 Hz, 3H), 1.25-1.62 (m, 12H), 2.70 (t, J = 7.8 Hz, 2H), 6.06 (br. s, 2H), 6.46-6.58 (m, 11H), 6.99 (br. s, 2H) ppm. ^{13}C NMR (125 MHz, C_6D_6): δ 14.3, 23.0, 25.5, 29.7, 29.81, 29.83, 30.2, 32.3, 105.8, 105.9, 107.5, 107.7, 107.86, 107.88, 107.92, 107.98, 108.0, 108.1, 108.2, 109.9, 111.7, 125.1, 141.6, 142.2, 145.3, 145.7, 145.8, 146.0, 146.1, 146.21, 146.24, 146.5, 146.6, 146.69, 146.74, 146.8 ppm. UV-Vis (CH_2Cl_2): λ_{max} = 410 nm. HRMS (FD): m/z calcd for $C_{36}H_{32}O_7$: 576.2148; found 576.2156.

Synthesis of 3'''',4'''-dioctyl-2,2':5',2'':5'',2''':5''',2'''':5'''',2''''':5''''', 2'''''':5'''''',2'''''''-octifuran (5). A mixture of 5,5'-dibromo-3,3'-dioctyl-2,2'-bifuran (**16**) (0.08 g, 0.15 mmol), [2,2':5',2''-terfuran]-5-yltributylstannane (**11**) (0.15 g, 0.31 mmol), and $Pd(PPh_3)_4$ (0.01 g, 0.008 mmol) in 35 mL dry toluene was refluxed for 24 h under N_2. After evaporation of toluene, the mixture was quenched with water, extracted with hexane, dried ($MgSO_4$), and evaporated. Flash chromatography on a silica column, using hexane as eluent gave **5** as an orange-colored product (0.04 g, 34% yield). ^{1}H NMR (500MHz, $CDCl_3$): δ 0.87 (t, J = 7.0 Hz, 6H), 1.18-1.49 (m, 20H), 1.66-1.75 (m, 4H), 2.79 (t, J = 7.6 Hz, 4H), 6.49 (dd, J = 3.4, 1.7 Hz, 2H), 6.60 (d, J = 3.6 Hz, 2H), 6.64 (br, 6H), 6.68 (d, J = 3.5 Hz, 2H), 6.70 (d, J = 3.5 Hz, 2H), 7.44 (br, 2H) ppm. ^{13}C NMR (125 MHz, $CDCl_3$): δ 14.3, 22.9, 25.5, 29.5, 29.7, 29.8, 30.5, 32.1, 105.7, 107.1, 107.3, 107.4, 107.6, 109.4, 111.7, 125.0, 142.1, 142.2, 144.5, 145.5, 145.6, 146.08, 146.11, 146.4 ppm. HRMS (FD): *m/z* calcd for $C_{48}H_{50}O_8$: 753.3506; found 754.3511

Synthesis of 3,3',3''-trimethyl-2,2':5',2''-terfuran (6). To a flask containing 40 mL dry ether, 3,3',3''-tribromo-2,2':5',2''-terfuran (**14**) (0.53 g, 1.21 mmol) and $Ni(dppp)Cl_2$ (0.06 g, 0.11 mmol) at 0°C, was added with a syringe, a solution of methylmagnesium bromide (1.6 mL of 3M solution, 4.80 mmol). The reaction mixture was stirred for 30 min at the same temperature. The ice-bath was removed and the reaction mixture was allowed to attain room temperature. The reaction mixture was refluxed for 24 h before being poured into ice-water. The product was then extracted with ether and the combined organic layers were washed with brine and dried over $MgSO_4$. The solvent was evaporated in vacuum. The compound was purified by column chromatography (elution with hexane) to give the terfuran **6** as a colorless oil which solidified on cooling at −20°C (0.15 g, 51% yield). ^{1}H NMR (300 MHz, $CDCl_3$): δ 2.27 (s, 3H), 2.30 (s, 6H), 6.30 (d, J = 1.7 Hz, 1H), 6.31 (d, J = 1.8 Hz, 1H), 6.40 (s, 1H), 7.31 (d, J = 1.7 Hz, 1H), 7.37 (d, J = 1.7 Hz, 1H) ppm. ^{13}C NMR (100 MHz, $CDCl_3$): δ 10.86, 10.9, 11.0, 109.9, 114.4, 114.7, 116.5, 117.1, 118.8, 141.1, 141.2, 141.9, 142.3, 143.0, 145.7 ppm. UV-Vis (CH_3CN): λ_{max} = 329 nm. HRMS (FD): m/z calcd for $C_{15}H_{14}O_3$: 242.0943; found 242.0936.

Synthesis of 3,3',3''',4''-tetramethyl-2,2':5',2'':5'',2'''-quaterfuran (7). Dry THF (15 mL) and a hexane solution of 5-bromo-3,3'-dimethyl-2,2'-bifuran (**18**) (0.25 g, 1.04 mmol) were added to a 100 mL round-bottomed 3-necked

flask. A temperature of -78°C was maintained and n-BuLi (0.75 mL of 1.6 M solution, 1.20 mmol) was added with a syringe. The reaction mixture was stirred at -78°C for 2 h. $CuCl_2$ (0.16 g, 1.20 mmol) was added in one portion and the reaction mixture was allowed to reach room temperature and stirred for 8 h. The mixture was quenched with water, extracted with ether, dried in $MgSO_4$, and evaporated. Flash chromatography on a silica column using hexane as eluent gave **7** (0.02 g, 13% yield) as a pale yellow powder. ^{1}H NMR (300 MHz, C_6D_6): δ 2.20 (s, 6H), 2.25 (s, 6H), 6.04 (d, *J* = 1.8 Hz, 2H), 6.39 (s, 2H), 7.04 (d, *J* = 1.8 Hz, 2H) ppm. ^{13}C NMR (125 MHz, C_6D_6): δ 10.7, 10.8, 110.0, 114.5, 117.4, 119.0, 141.3, 142.8, 143.4, 145.0 ppm. UV-Vis (CH_2Cl_2): λ_{max} = 360 nm. HRMS (FD): m/z calcd for $C_{20}H_{18}O_4$: 322.1205; found 322.1210.

Synthesis of 2,5-dibromo-3-octylfuran (12).[2] Into a solution of 3-octylfuran[2] (0.10 g, 0.55 mmol) in benzene (2.5 mL) was added *N*-bromosuccinimide (0.22 g, 1.24 mmol), and the mixture was stirred for 2 h in the dark at room temperature. The mixture was then extracted with hexane, washed with a saturated solution of sodium bicarbonate and brine, dried ($MgSO_4$), and evaporated. Flash chromatography using hexane as eluent gave the title product (0.13 g, 68% yield). ^{1}H NMR (300 MHz, $CDCl_3$): δ 0.87 (t, *J* = 6.6 Hz, 3H), 1.25-1.51 (m, 12H), 2.29 (t, *J* = 7.5 Hz, 2H), 6.21 (s, 1H) ppm. ^{13}C NMR (125 MHz, $CDCl_3$) δ 14.3, 22.8, 25.4, 29.2, 29.3, 29.4, 29.5, 32.0, 114.9, 119.7, 121.4, 127.6 ppm. HRMS (FD): *m/z* calcd for $C_{12}H_{18}Br_2O$: 335.9724; found 335.9736.

Synthesis of 3,3'-dibromo-2,2'-bifuran (13).[3] A solution of lithium diisopropylamide (LDA) (17.2 mL of 2 M LDA in THF/heptane/ethylbenzene, 34.4 mmol) was added dropwise to a solution of 3-bromofuran (5.0 g, 34.0 mmol) in dry tetrahydrofuran (50 mL) and dry ether (50 mL) at −78°C under N_2. The reaction mixture was kept at −78°C and stirred for 1 h. $CuCl_2$ (4.6 g, 34.0 mmol) was added in one portion and the reaction mixture was allowed to reach room temperature and stirred overnight. The mixture was quenched with water, extracted with hexane, dried in $MgSO_4$, and evaporated. Flash chromatography on a silica column using hexane as eluent gave **13** (2.7 g, 54% yield) as a white crystalline product. ^{1}H NMR (400MHz, $CDCl_3$): δ 6.55 (d, *J* = 1.9 Hz, 2H), 7.47 (d, *J* = 1.9 Hz, 2H) ppm. ^{13}C NMR (100 MHz, $CDCl_3$) δ 99.2, 115.8, 141.7, 143.4 ppm. HRMS (FD): *m/z* calcd for $C_8H_4Br_2O_2$: 291.8558; found 291.8564.

Synthesis of 3,3',3''-tribromo-2,2':5',2''-terfuran (**14**). Compound **14** was obtained as a minor product with a 6% yield during the synthesis of the precursor 3,3'-dibromo-2,2'-bifuran (**13**). ^{1}H NMR ($CDCl_3$, 300 MHz): δ 6.55 (d, *J* = 1.9 Hz, 1H), 6.57 (d, *J* = 1.9 Hz, 1H), 6.99 (s, 1H), 7.43 (d, *J* = 1.9 Hz, 1H), 7.50 (d, *J* = 1.9 Hz, 1H) ppm. ^{13}C NMR (100 MHz, $CDCl_3$): δ 97.6, 99.6, 100.5, 112.9, 115.99, 116.02, 141.0, 141.5, 141.8, 143.1, 143.6, 144.8 ppm. HRMS (FD): m/z calcd for $C_{12}H_5BrO_3$: 435.7768; found 435.7754.

Synthesis of 3,3'-dioctyl-2,2'-bifuran (15). To a solution of 3,3'-dibromo-2,2'-bifuran (0.50 g, 1.70 mmol) and Ni(dppp)Cl_2 (0.20 g, 0.37 mmol) in dry ether (25 mL) at 0ºC under N_2 was added dropwise a solution of octylmagnesium bromide (3.4 mL of 2M solution in ether, 6.8 mmol). The reaction mixture was refluxed overnight. The mixture was quenched with water, extracted with hexane, dried ($MgSO_4$), and evaporated. Flash chromatography on a silica column using hexane as eluent gave **15** (0.45 g, 74% yield). ^{1}H NMR (500MHz,

$CDCl_3$): δ 0.87 (t, *J* = 6.8 Hz, 6H), 1.20-1.40 (m, 20H), 1.50 – 1.58 (m, 4H), 2.60 (t, *J* = 7.7 Hz, 4H), 6.33 (d, *J* = 1.2 Hz, 2H), 7.35 (d, *J* = 1.4 Hz, 2H) ppm. ^{13}C NMR (125 MHz, $CDCl_3$): δ 14.3, 22.9, 25.1, 29.46, 29.55, 29.6, 30.5, 32.1, 112.8, 123.0, 141.3, 142.3 ppm. HRMS (FD): m/z calcd for $C_{24}H_{38}O_2$: 358.2872; found 358.2883.

Synthesis of 5,5'-dibromo-3,3'-dioctyl-2,2'-bifuran (16). N-Bromosuccinimide (0.21 g, 1.20 mmol) was added to a solution of 3,3'-dioctyl-2,2'-bifuran (0.20 g, 0.56 mmol) in 30 mL benzene at room temperature under N_2. The reaction mixture was kept at room temperature and stirred for 2 h. The mixture was quenched with saturated sodium bicarbonate and saturated sodium thiosulfate pentahydrate ($Na_2S_2O_3 \cdot 5H_2O$). The product was extracted with hexane, dried ($MgSO_4$), and evaporated. Flash chromatography on a silica column using hexane as eluent gave **16** (0.23 g, 80% yield) as a white crystalline product. ^{1}H NMR (300MHz, C_6D_6): δ 0.92 (t, *J* = 6.8 Hz, 6H), 1.15-1.33 (m, 20H), 1.35 – 1.47 (m, 4H), 2.48 (t, *J* = 7.6 Hz, 4H), 5.94 (s, 2H) ppm. ^{13}C NMR (125 MHz, C_6D_6): δ 14.4, 23.1, 25.1, 29.5, 29.6, 29.7, 30.2, 32.2, 114.7, 121.5, 126.6, 143.6 ppm. HRMS (FD): m/z calcd for $C_{24}H_{36}Br_2O_2$: 514.1082; found 514.1091.

Synthesis of 3,3'-dimethyl-2,2'-bifuran (17). To a 250 ml 3-necked round-bottomed flask was added dry ether (100 mL) and degassed with nitrogen. To it were added 3,3'-dibromo-2,2'-bifuran (3.02 g, 10.3 mmol), Ni(dppp)Cl_2 (0.43 g, 0.8 mmol), and methylmagnesium bromide (10.3 mL of 3M solution, 31.0 mmol) at 0°C and the mixture was stirred for 30 min at the same temperature. The ice-bath was removed and the reaction mixture was allowed to attain room temperature. The reaction mixture was refluxed for 24 h before being poured into ice-water. The product was then extracted with ether and the combined organic layers were washed with brine and dried over $MgSO_4$. The solvent was evaporated in vacuum. The compound was purified by column chromatography (elution with hexane) to give bifuran **17** as a pale yellow oil (1.2 g, 71% yield). ^{1}H NMR (300 MHz, $CDCl_3$): δ 2.22 (s, 6H), 6.30 (d, *J* = 1.8 Hz, 2H), 7.36 (d, *J* = 1.7 Hz, 2H) ppm. ^{13}C NMR (75 MHz, $CDCl_3$): δ 10.8, 114.2, 117.4, 141.3, 142.8 ppm. HRMS (FD): m/z calcd for $C_{10}H_{10}O_2$: 162.0681; found 162.0690.

Synthesis of 5-bromo-3,3'-dimethyl-2,2'-bifuran (18). To a solution of 3,3'-dimethyl-2,2'-bifuran (**17**) (0.39 g, 2.39 mmol) in tetrahydrofuran (15 mL) was added *N*-bromosuccinimide (0.41 g, 2.30 mmol), and the mixture was stirred for 3 h at 0°C. The mixture was then extracted with ether, washed with a saturated solution of sodium bicarbonate and brine, dried ($MgSO_4$), and evaporated. Flash chromatography using hexane as eluent gave the title product **18** (0.12 g, 21% yield). It was stored as a solution in hexane. ^{1}H NMR (300 MHz, C_6D_6): δ 1.96 (s, 3H), 2.04 (s, 3H), 5.81 (s, 1H), 5.93 (d, *J* = 1.8 Hz, 1H), 6.96 (d, *J* = 1.7 Hz, 1H) ppm.

Methods

Electrochemical polymerization and measurements were performed using a Bio-Logic SAS VSP or Princeton Applied Research 263A potentiostat in a standard one compartment three-electrode setup. Spiral Pt-wire served as the counter electrode and an AgCl coated Ag wire, which was directly dipped into the electrolyte solution, was the pseudo reference electrode (+0.05 V vs. SCE). Ag/AgCl-wire was prepared by dipping silver wire for 1 min in a solution of 0.25 M $FeCl_3$ in 0.5M HCl. Pt-wire counter electrode were cleaned by flame followed by dipping in concentrated sulfuric acid. Number of working electrodes were used: indium tin oxide (ITO)-coated glass slides ($7 \times 50 \times 0.7$ mm^3, Rs = 5‑15 Ω/□, Delta Technologies, Stillwater); 2 mm diameter Pt-disk (CH Instruments, Inc.); Au-coated quartz crystal (CH Instruments, Inc., see SI); a highly oriented pyrolytic graphite (HOPG) 10×10 mm^2 piece (NT-MDT Co., ZYB quality), and; a gold interdigitated array (IDA) microelectrode (ABTECH Scientific, Inc.). ITO-coated glass slides were cleaned by washing with ethanol and acetone. Fresh surface of HOPG electrode was obtained by removal of the top layer with Scotch adhesive tape. The Pt-disk electrode was polished to a mirror finish with alumina gel (0.3 and 0.05 μm) on microcloth pads. An ACN solution containing 0.1 M tetrabutylammonium trifluoromethanesulfonate ($TBACF_3SO_3$) used as the electrolyte unless stated otherwise.

The spectroelectrochemical setup consisted of a quartz cuvette as the electrochemical cell in a custom-made holder, an ITO-coated glass electrode used as the working electrode, Ag/AgCl-wire used as a pseudo reference electrode, and Pt-wire used as a counter electrode. Potentials were applied using a Princeton Applied Research 263A potentiostat. Absorption spectra were taken in a quartz cuvette with 10 mm optical path length (100-QX, Hellma) with a JASCO V-570 UV-Vis-NIR spectrophotometer.

FTIR-ATR spectroscopy was performed using a Nicolet 6700 FT-IR (Thermo Fisher Scientific Inc.) equipped with a MIRacle (PIKE Technologies) ATR sampling accessory with a ZnSe crystal using an MCT/A liquid nitrogen cooled detector. The presented spectra were not corrected for ATR intensities.

Scanning electron microscopy (SEM) images were recorded using a Leo Ultra 55 FEG SEM with an operating voltage of 3 keV. AFM topography images were acquired using a P47 AFM (NT-MDT) equipped with a small scanner. Images were recorded in tapping mode in the air at room temperature (20‑23°C) using silicon micro cantilevers (OMCL-AC240TS-W2, Olympus). The set point ratio was adjusted to 0.75–0.8 (corresponding to "light" tapping) and the scan rate was set to 1 Hz. Imaging was carried out in different scan directions and at different scales to verify the consistency and robustness of the evaluated structures. The thickness of polymer films was measured by AFM profilometry.

In situ conductivity

In situ conductivity measurements were performed on a Bio-Logic SAS VSP potentiostat equipped with two independent channels using an IDA (interdigitated array) microelectrode. The IDA electrode is consisting of two arrays of 50 gold digits (strips), each 10 µm wide and 5 mm long with an interdigit distance of 10 µm (Figure S1), where only the microarrays are accessible to the electrolyte solution. The first channel of the potentiostat was used in the standard three electrode mode using Pt-wire as the counter electrode, Ag/AgCl-wire as the reference electrode, and one of the arrays of the IDA electrode as the working electrode (W1 in Figure S2). A second channel was used to supply a small constant probe potential of 10 mV between two set of digits (working electrodes W1 and W2, Figure S2). Both channels were synchronized and voltammetric (CV) and probe current data were acquired simultaneously. Polymer films were grown potentiodynamicaly or potentiostatically. During electropolymerization, the increasing conductance of the growing film was monitored. It was noticed, that conductance saturates reaching some value. Therefore, the polymerization was stopped when the increase in conductance was small (less than 10%). Once grown, the films were washed with ACN and then placed in monomer-free electrolyte solutions. We found that using such setup low conductance values ($<10^{-2}$ S) could not be measured reliably because of the small negative contribution from faradic current. Therefore the on-off ratio cannot be extracted from the experimental data.

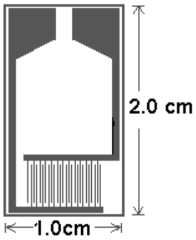

Figure S1. IDA electrode design (adapted from technical data sheet, ©ABTECH Scientific, Inc., http://www.abtechsci.com/pdfs/tds1050.pdf)

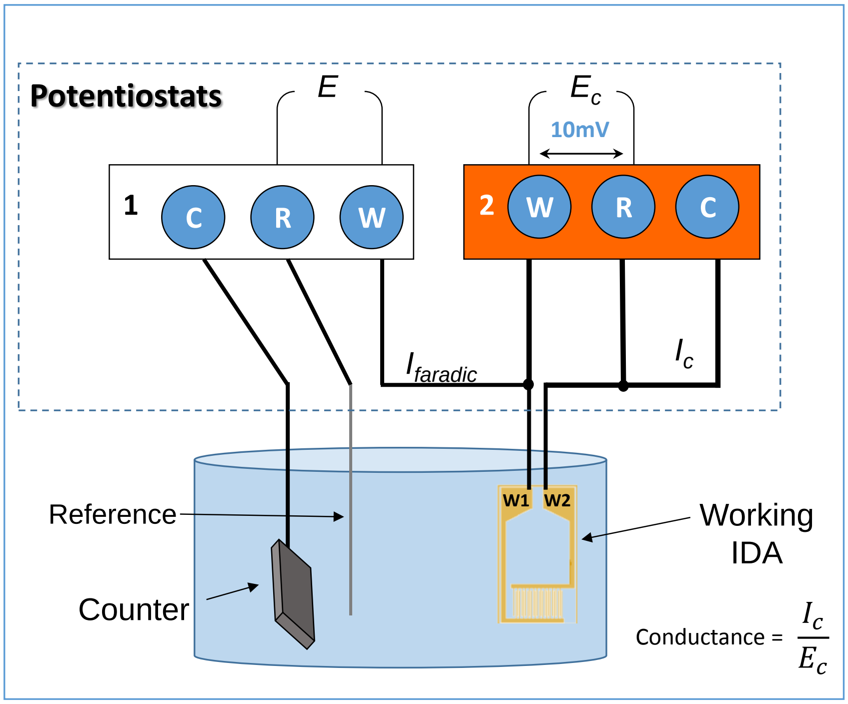

Figure S2. Connections setup for in situ conductivity measurements.

Calculations

Computational studies were carried out using density functional theory (DFT) with the Gaussian 09 series of programs.[4] The geometries of the polymers were fully optimized using the periodic boundary conditions (PBC) formalism and hybrid DFT with Becke's three-parameter exchange functional combined with the LYP correlation functional (B3LYP) and the 6-31G(d) basis set (PBC/B3LYP/6-31G(d)).[5] The PBC/B3LYP/6-31G(d) level generally provides a good estimation of the optical band gaps of conjugated polymers.[6] Vibrational frequencies were scaled by 0.96.[7] An ultrafine integration grid was employed (integral=ultrafine) for all calculations.

Additional Data

Electrochemical polymerization

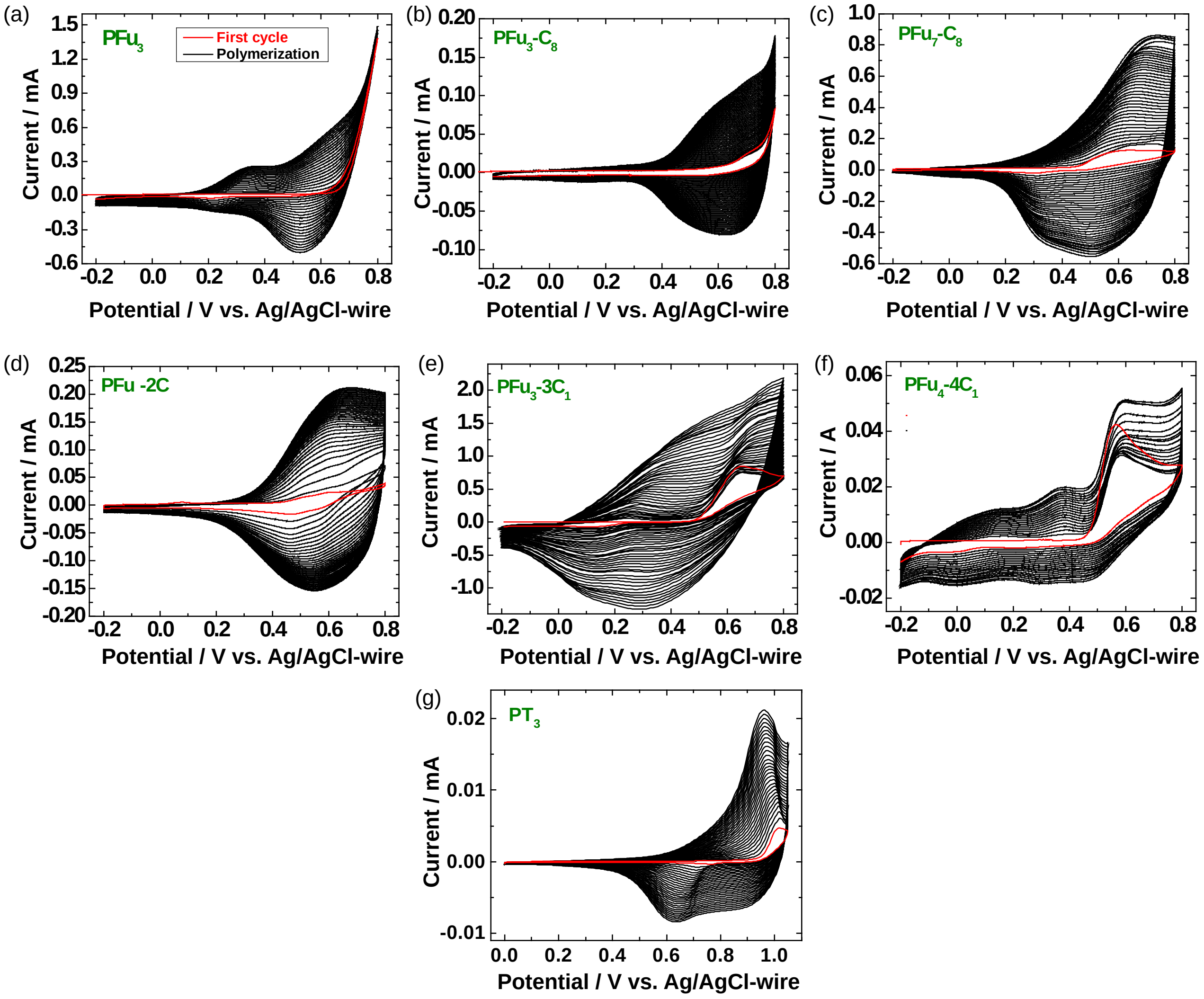

Figure S3. Potentiodynamic electropolymerization of monomer/oligomer to polymer (a) **P1**, (b) **P2**, (c) **P4**, (d) **P5**, (e) **P6**, (f) **P7** and (g) PT_3 in 0.1 M $TBACF_3SO_3$/ACN on ITO coated glass.

Table S1. Onset and peak oxidation potentials of oligofurans **1-7** and terthiophene (T_3) as measured from the first CV wave during electropolymerization.

Oligomer	E_{ox},[a] V vs. Ag/AgCl-wire	E_p,[b] V vs. Ag/AgCl-wire	E_p,[c] V vs. Ag/AgCl-wire
Fu_3 (**1**)	0.62	>0.8	>0.8
Fu_3-C_8 (**2**)	0.51	~0.7[d]	0.68
Fu_5-C_8 (**3**)	0.43	0.61	0.58
Fu_7-C_8 (**4**)	0.40	~0.6[d]	0.54
Fu_8-2C_8 (**5**)	0.37	~0.5[d]	0.50
Fu_3-3C_1 (**6**)	0.44	0.65	0.61
Fu_4-4C_1 (**7**)	0.41	0.56	0.53
T_3	0.95	1.01	1.00

[a]Onset oxidation potential; [b]Peak oxidation potential; [c]Peak oxidation potential from semiderivative plot; [d]Peak is not resolved.

Table S2. Optimized electropolymerization conditions for **P1** and **P3**.

Polymer	Electrode	Electropolymerization conditions (vs. Ag/AgCl-wire)
**P1**	ITO	1 mM Fu_3 in 0.1 M $TBACF_3SO_3$/ACN<br>Constant potential of 0.78V for 10-20 mC
**P1**	Gold IDA	1 mM Fu_3 in 0.1 M $TBACF_3SO_3$/ACN<br>Two step:<br>1) Constant potential of 0.8V for 0.3mC<br>2) CV from -0.2V to 0.85V at 50 mV s^{-1}
**P3**	ITO	0.1-0.3 mM Fu_5-C_8 in 0.1 M $TBACF_3SO_3$/ACN<br>CV from -0.2V to 0.8V at 50 mV s^{-1}
**P3**	Gold IDA	0.1-0.3 mM Fu_5-C_8 in 0.1 M $TBACF_3SO_3$/ACN<br>CV from -0.2V to 0.8V at 50 mV s^{-1}
**P3**	HOPG	0.1-0.3 mM Fu_5-C_8 in 0.1 M $TBACF_3SO_3$/CAN<br>CV from -0.2V to 0.78V at 50 mV s^{-1}

Electrolyte Effect

Polyfurans prepared in 0.1 M $TBABF_4$/ACN and 0.1 M $TBAClO_4$/ACN electrolyte showing almost no optical activity with blue-shifted maximum absorption peak, meaning that non-conjugated polyfuran is obtained (Figure S4).

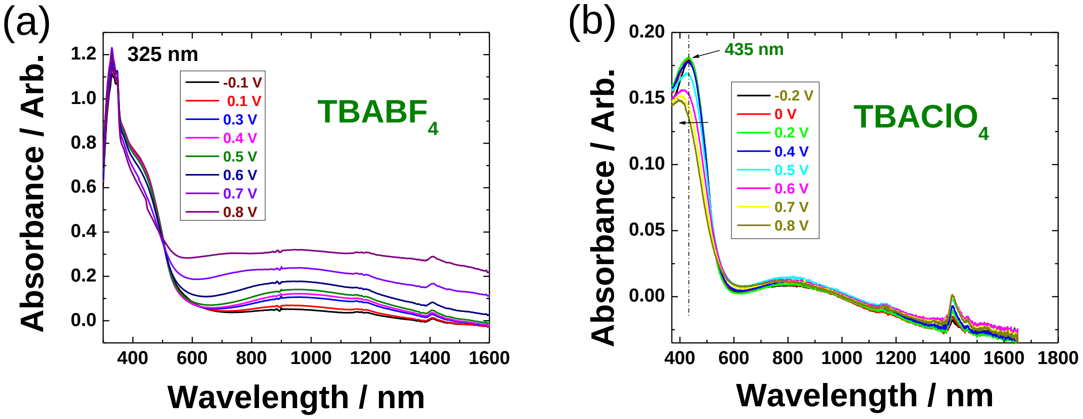

Figure S4. Spectroelectrochemistry of **P1** (PFu_3) prepared in ACN with (a) 0.1 M $TBABF_4$ and (b) 0.1 M $TBAClO_4$.

CV of polymers

The CV of **P1** clearly shows a two-step oxidation process (Figure 2a). For **P2**, **P3** and **P7**, however, the same two-step oxidation, which is similar to that observed previously in polythiophenes,[8] is less resolved (Figure S5, S6).

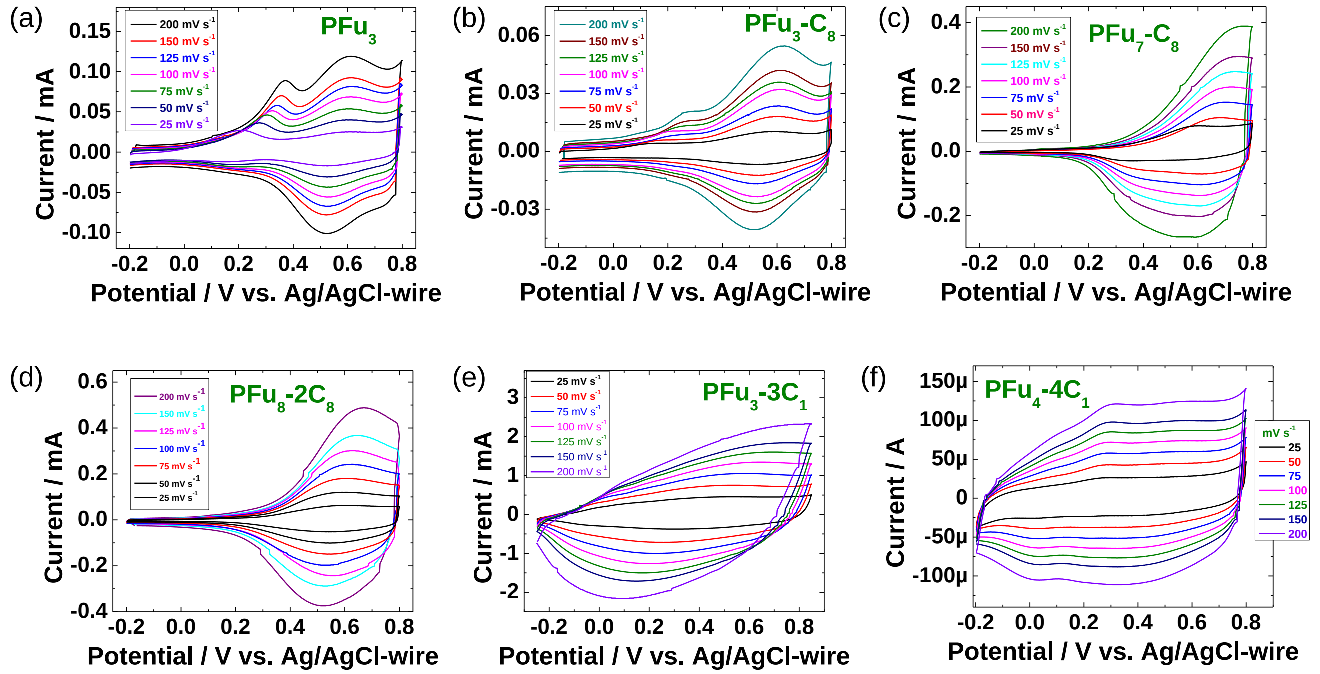

Figure S5. Monomer-free cyclic voltammograms of (a) **P1**, (b) **P2**, (c) **P4**, (d) **P5**, (e) **P6** and (f) **P7** in TBACF$_3$SO$_3$/ACN on ITO glass electrode. The electrodeposition and monomer-free electrochemistry measurements were performed in the same electrolyte.

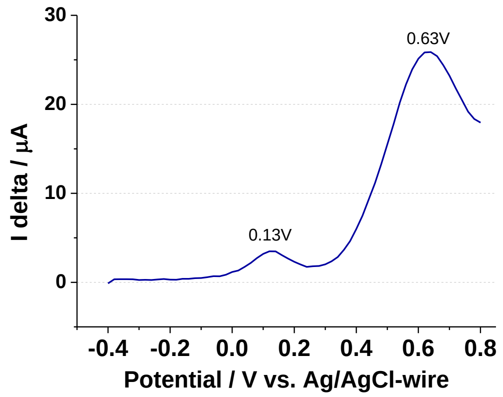

Figure S6. Monomer-free square-wave voltammograms of **P3** in TBACF$_3$SO$_3$/ACN on Pt-disk electrode showing a two-step oxidation (SWV parameters: P_H=50 mV, P_W=200 ms, S_H=20 mV).

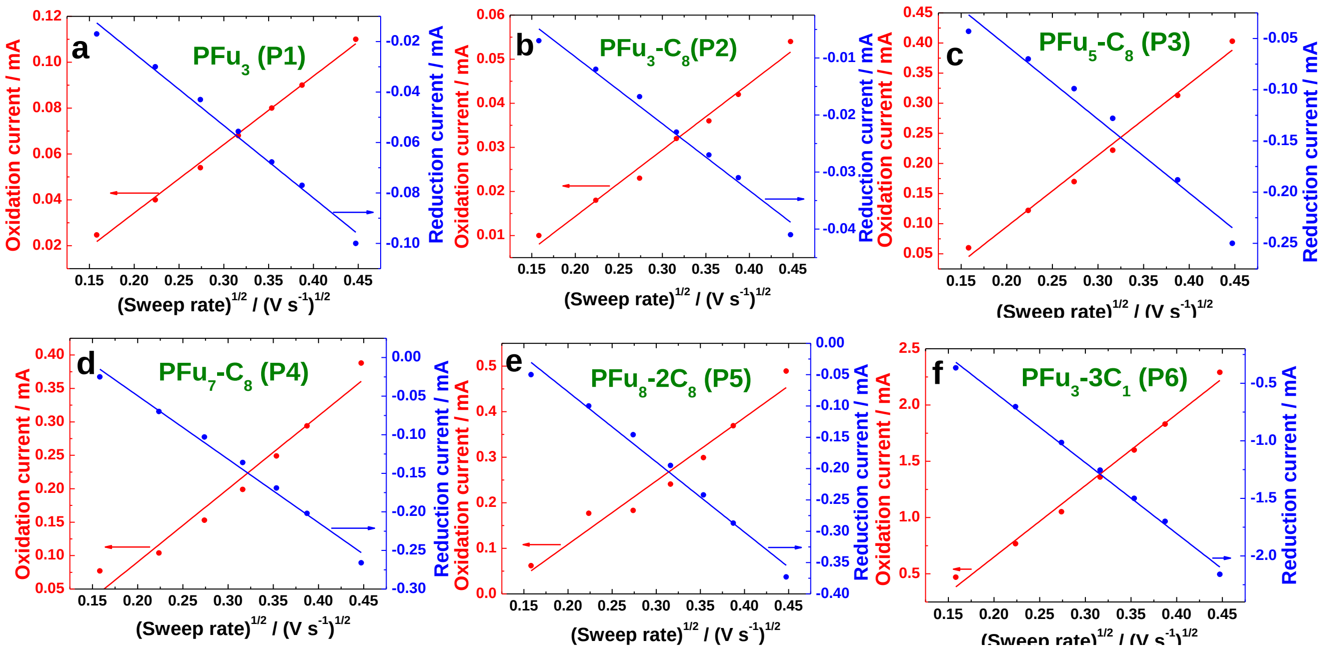

Figure S7. The current vs. (sweep rate)$^{1/2}$ of (a) **P1**, (b) **P2**, (c) **P3**, (d) **P4**, (e) **P5** and (f) **P6**.

Spectroelectrochemistry

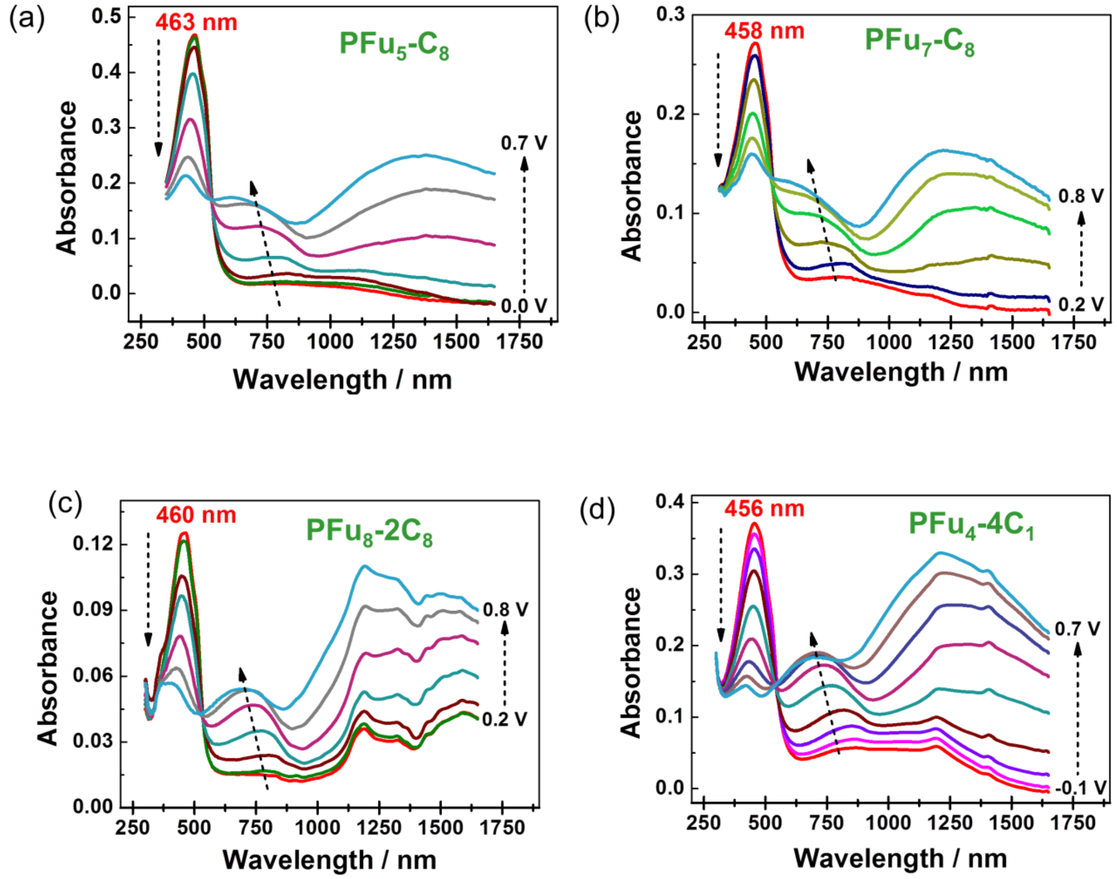

Figure S8. Spectroelectrochemistry of polymer films (a) **P3**, (b) **P4**, (c) **P5** and (d) **P7** on ITO coated glass electrode in monomer-free 0.1 M $TBACF_3SO_3$/ACN electrolyte.

DFT calculations

The effect of alkyl substitution of polyfurans on crystal orbital energies, band gaps (E_g), and geometries was studied by PBC/B3LYP/6-31G(d) calculations. Calculations of methyl substituted polyfurans, which serve as models, show that increase of the degree of methyl substitution on the polyfuran backbone systematically decreases HOCO, LUCO and E_g energies (Table S2). Moreover, E_g energies are linearly decrease as the methyl substitution ratio (i.e. the average number of methyl substituents per furan ring) increases (Figure S9). This indicates that the methyl substitution of polyfurans is an additive property.

Table S3. The calculated (PBC/B3LYP/6-31G(d)) HOCO, LUCO and E_g energies (in eV) of model methyl substituted polyfurans.

**Methyl ratio**[a]	**Calculated repeating unit**	**HOCO (eV)**	**LUCO (eV)**	**E_g (eV)**
0.00	Fu_2	-4.359	-1.947	2.411
0.14	Fu_{14}-Me_2	-4.308	-1.919	2.388
0.17	Fu_6-Me	-4.300	-1.915	2.385
0.20	Fu_{10}-Me_2	-4.288	-1.909	2.379
0.33	Fu_6-Me_2	-4.243	-1.883	2.360
0.50	Fu_2-Me	-4.191	-1.852	2.338
1.00	rrFu_6-Me_6[b]	-4.027	-1.763	2.264

[a] Average number of methyl substituents per furan ring; [b] rr = regio-regular.

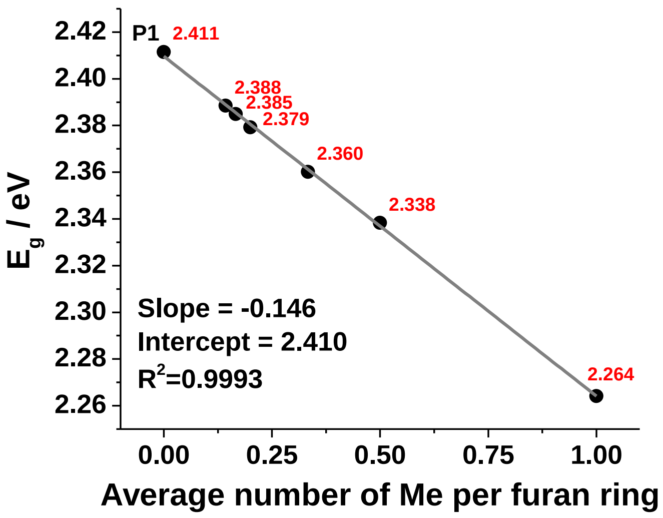

Figure S9. Linear dependence of the bandgap of polyfurans on their methyl substitution ratio.

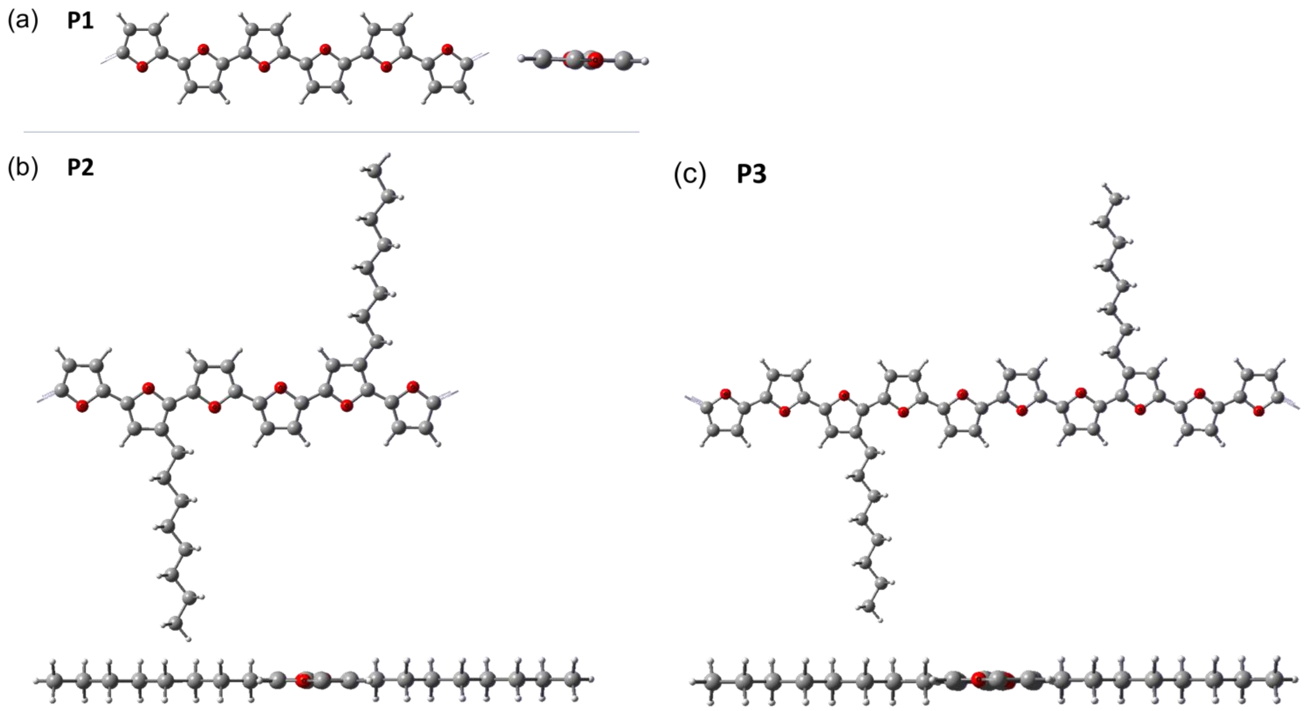

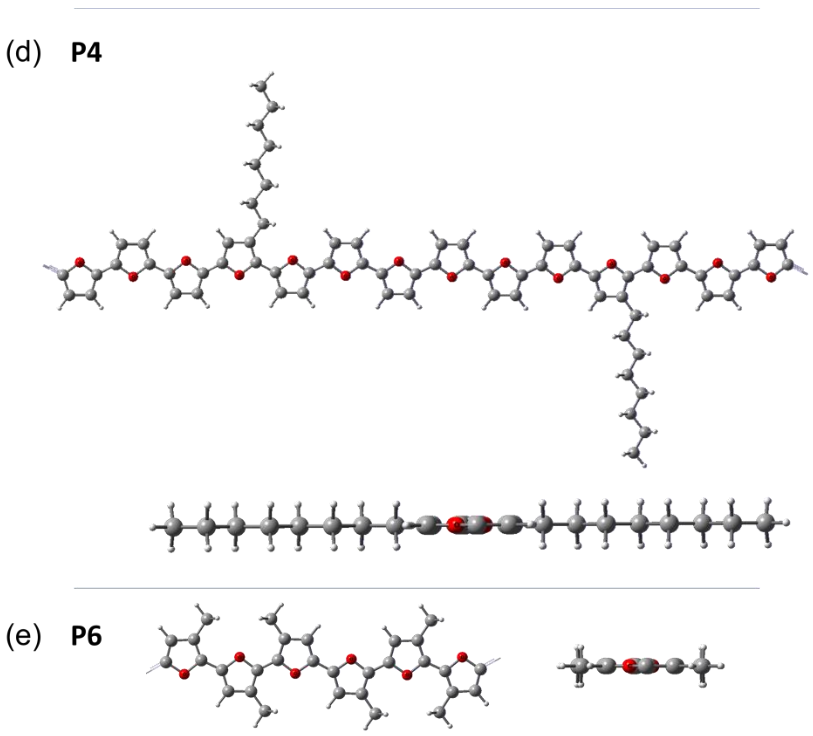

Figure S10. The side view and the view along the polyfuran backbone of DFT PBC/B3LYP/6-31G(d) calculated optimized geometry of (a) **P1**, (b) **P2**, (c) **P3**, (d) **P4**, and (e) **P6**.

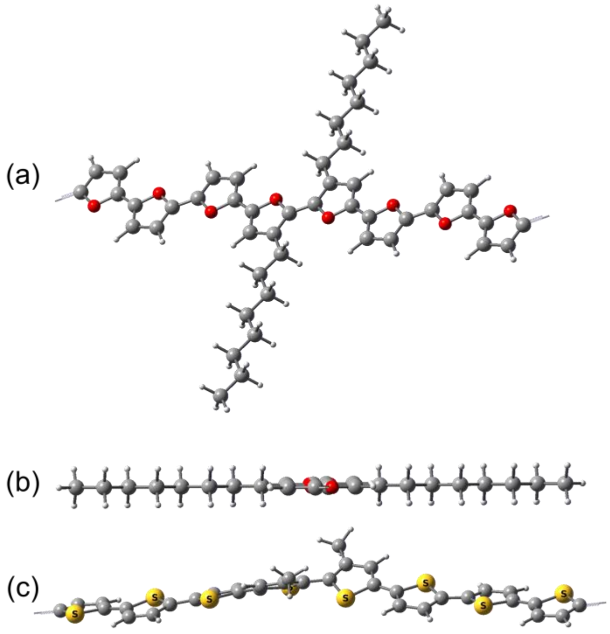

Figure S11. DFT PBC/B3LYP/6-31G(d) calculated optimized geometry of **P5**: (a) side view and (b) view along the polyfuran backbone. (c) Side view of the DFT calculated optimized geometry of the model thiophene analog of **P5**, PT_8-$2C_1$.

Table S4. List of DFT calculated (B3LYP/6-31G(d)// B3LYP/6-31G(d)) vibrational modes of Fu_{20} with IR intensities greater than 25 and frequencies greater than 700 cm^{-1}. Scaling by 0.96 according to NIST Computational Chemistry Comparison and Benchmark Database.[7]

**Mode #**	**Freq, cm^{-1}**	**Scaled freq, cm^{-1}**	**IR intens.**
156*	733	704	66
165	796	765	33
168	797	765	505
169	798	766	179
171	799	767	51
217	906	870	157
237*	1039	998	180
239	1044	1002	366
259	1122	1078	119
280	1236	1187	758
300*	1324	1271	101
306	1326	1273	31
339	1477	1418	171
355	1553	1491	87
357	1558	1495	2378
358*	1581	1517	319
379*	3268	3138	31
381	3278	3147	543

* Vibrational modes of terminal furan units which are not observed in the polymer.

Mode 168 γ(CH): 797 (765) cm^{-1}

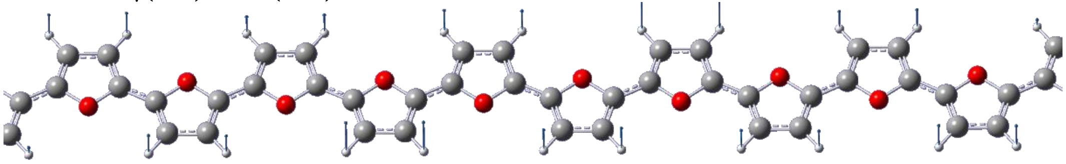

Mode 217 $\delta_{ring} + \nu_{ring}$(C-O): 906 (870) cm^{-1}

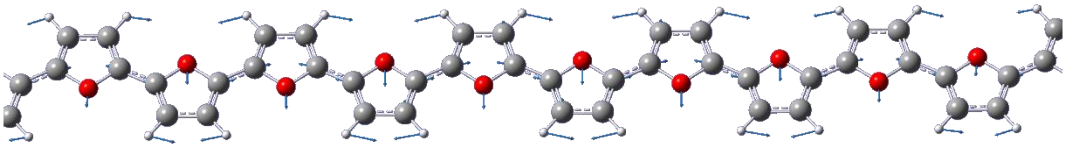

Mode 239 δ(CH)$_{ring}$: 1044 (1002) cm^{-1}

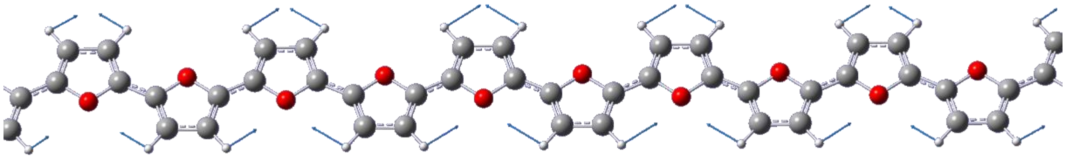

Mode 280 δ(CH)$_{ring}$ + ν(C-O)$_{ring}$: 1236 (1187) cm^{-1}

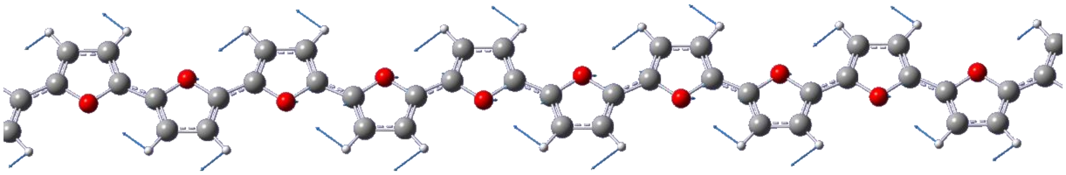

Mode 357 ν(C=C) + δ$_{ring}$: 1558 (1495) cm^{-1}

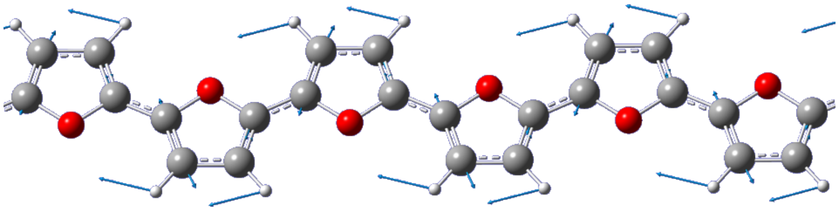

Mode 381 ν(CH)$_{ring}$: 3278 (3147) cm^{-1}

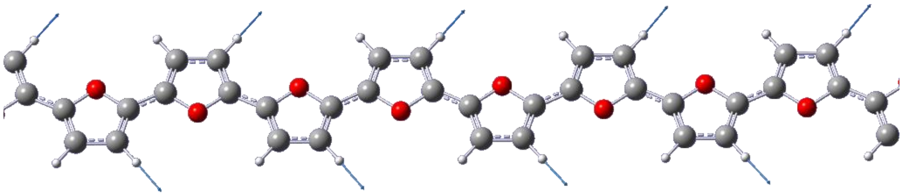

Figure S12. Vibrational displacements of the selected modes (B3LYP/6-31G(d)// B3LYP/6-31G(d)) of Fu$_{20}$. In parentheses given scaled frequencies.

Electrochemical Stability

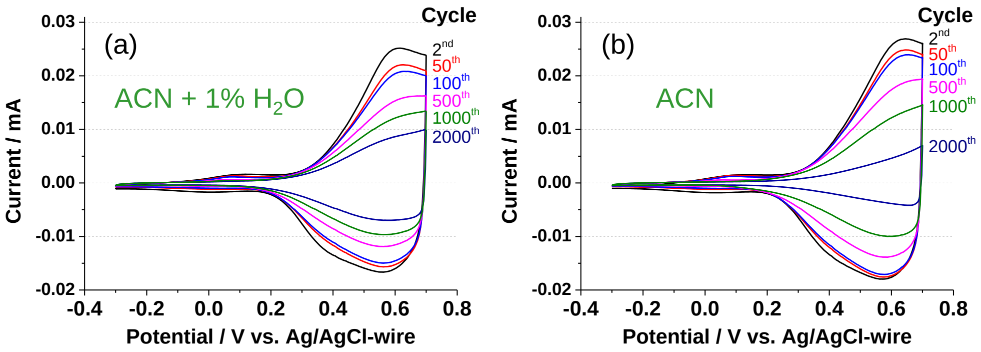

Figure S13. Long term stability study of **P3** in in monomer-free 0.1 M TBACF$_3$SO$_3$/ACN electrolyte, (a) with and (b) without addition of 1% H_2O to the electrolyte solution.

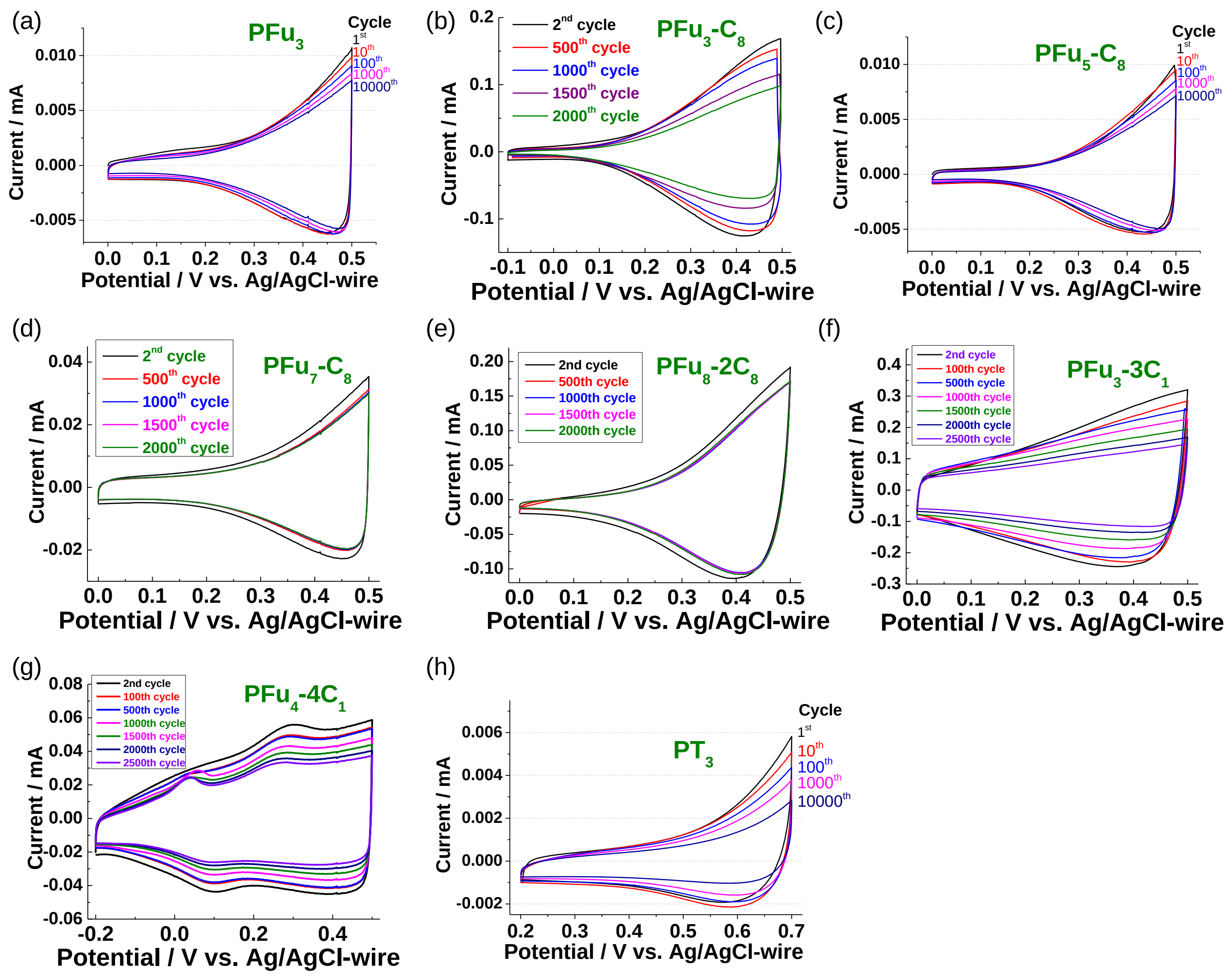

Figure S14. Long term stability study of (a) **P1**, (b) **P2**, (c) **P3**, (d) **P4**, (e) **P5**, (f) **P6**, (g) **P7** and (h) **PT$_3$** in the reduced potential range (up to 0.5 V). The electrodeposition and monomer-free electrochemistry were performed in the same electrolyte.

Conductivity measurements

A value of 10‑80 S/cm was reported by Tourillon and Garnier,[9] who first claimed to have prepared conjugated polyfuran via anodic electrochemical polymerization of furan. However, such high value seems to be unreliable, as Ohsawa et al.,[10] were unable to reproduce such a highly conductive polyfuran.

Nalwa[11] also reported conductivity as high as 20 S/cm. However, this value was measured on ill-defined samples as no characterization (other than EPR) was undertaken. As well, such high conductivity contradicts with his conclusion that that the polyfuran has only a low degree of conjugation.

In any case, the high voltage required for anodic coupling of furan should result in irreversible oxidation of the polymer backbone. Hence, in neither of these earlier reports were the conductivities measured on well-defined samples of polyfuran.

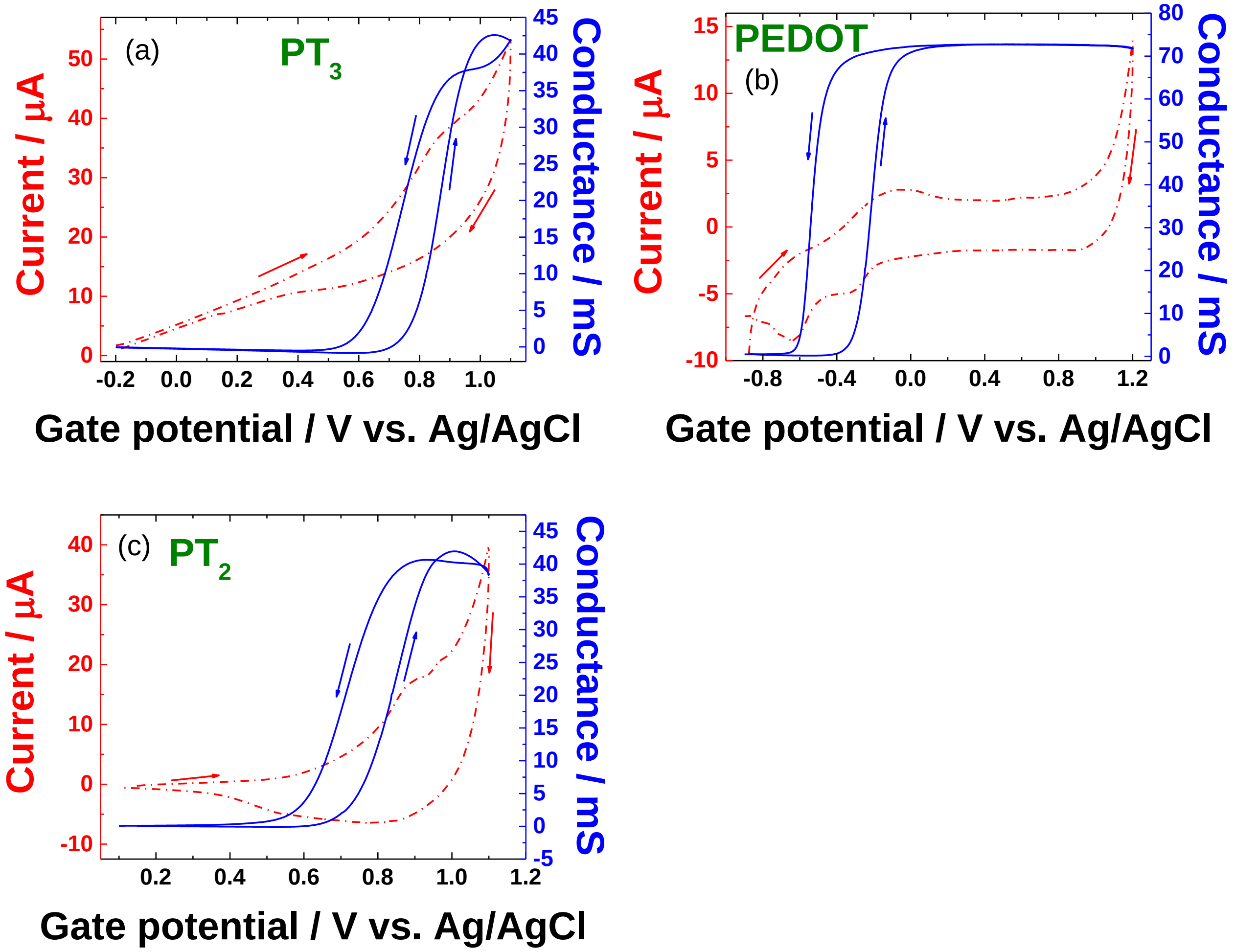

Figure S15. Cyclic voltammograms (dashed line) and in situ conductivity measurements (solid line) for (a) PT_3, (b) PEDOT and (c) PT_2 in monomer-free $TBACF_3SO_3$/ACN using a 10 μm interdigitated array gold microelectrode (50-digit). Sweep rates are 10 mV s^{-1} for cyclic voltammograms with a 10 mV probe potential for conductivity measurements.

IR Spectroscopy

Degradation study

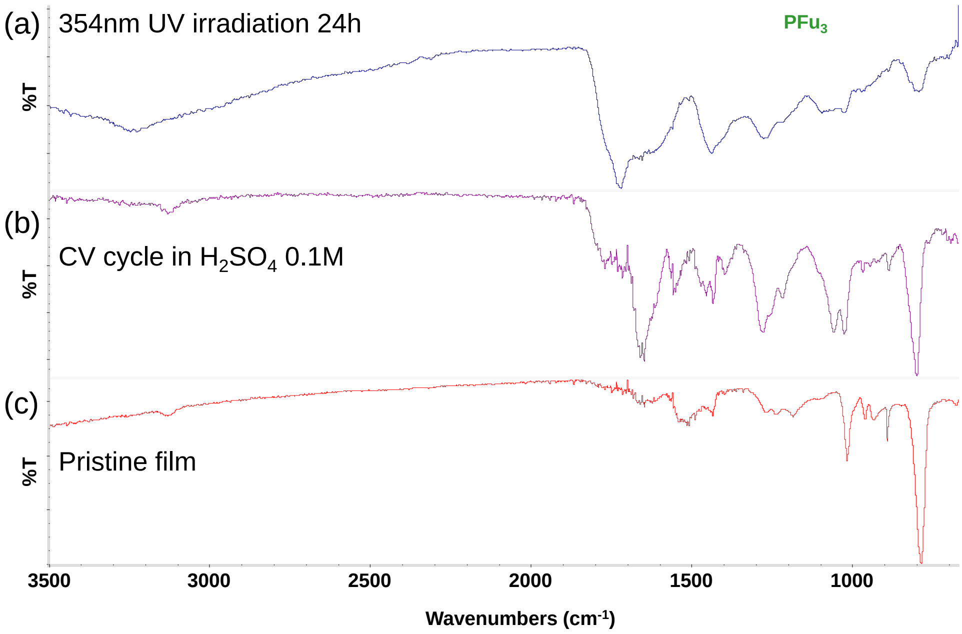

Figure S16. FTIR-ATR spectra of **P1** (PFu_3) film after (a) continuous UV irradiation at 354nm for 24h, (b) CV cycle from 0.0V to 1.0V vs. Ag/AgCl-wire in 0.1M H_2SO_4; (c) pristine film.

Morphology study

AFM data were processed using NT-MDT Nova[12] and Gwyddion[13] software.

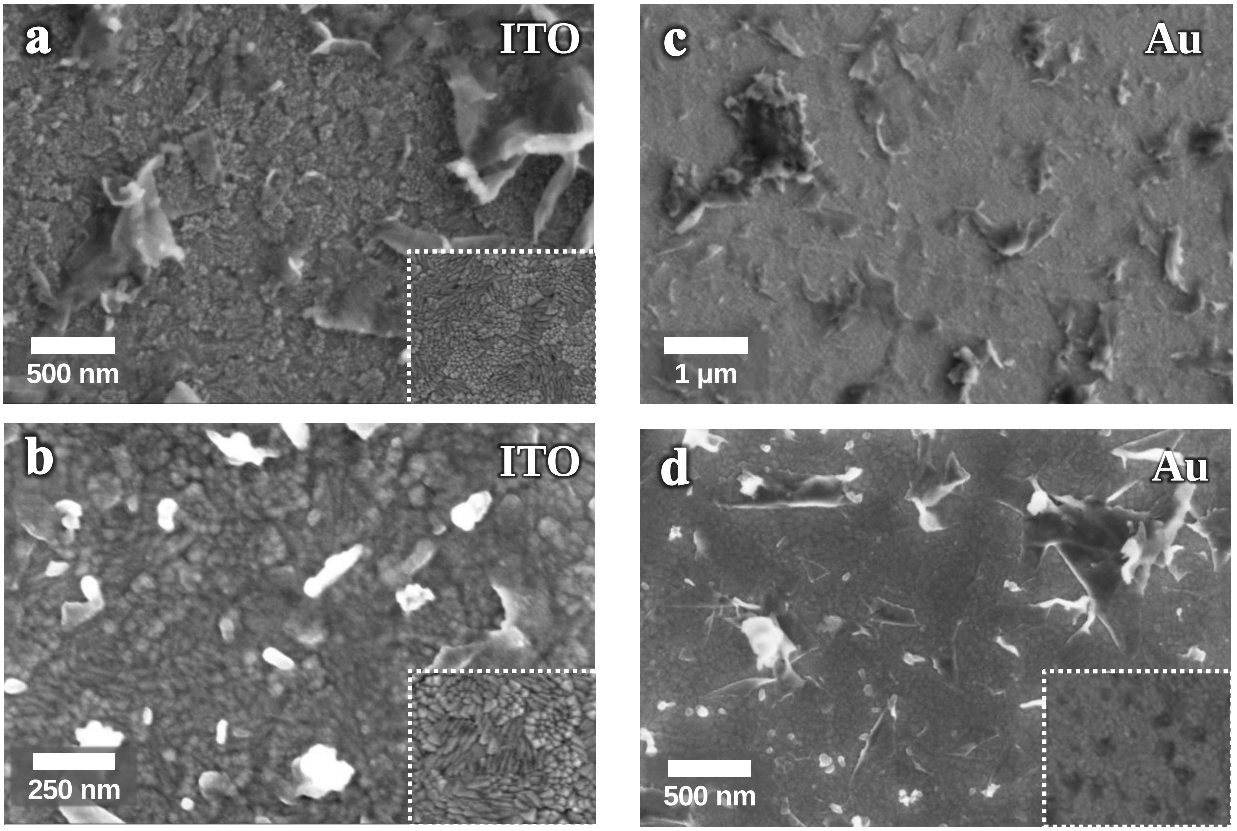

Figure S17. SEM images of PT_3 on (a,b) an ITO-coated glass and (c,d) an Au-coated glass electrode. Insets show clean electrodes (without polymer).

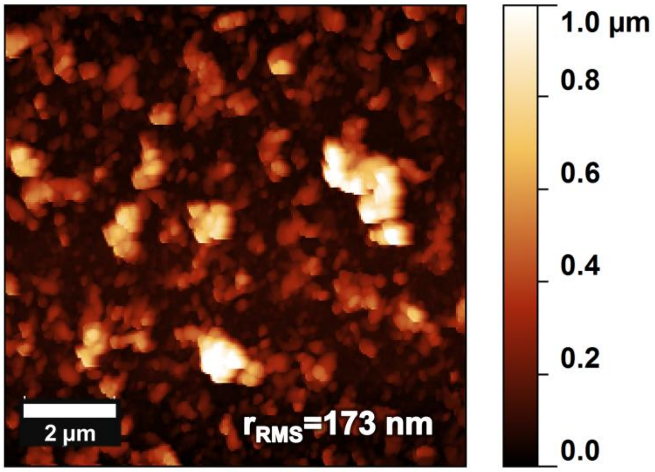

Figure S18. AFM image of PT_3 on an ITO electrode.

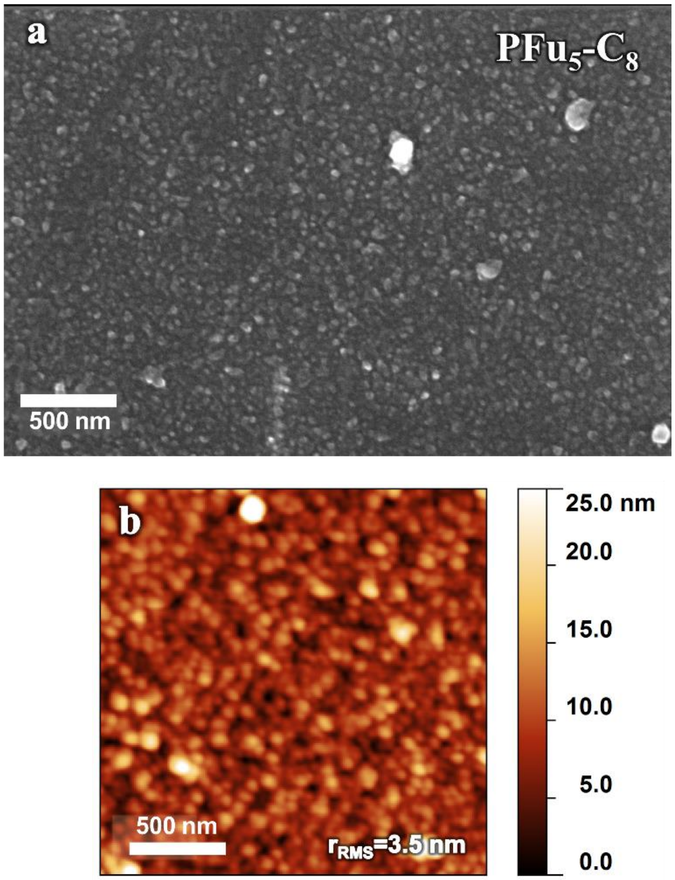

Figure S19. (a) SEM and (b) AFM images of **P3** (PFu_5-C_8) film prepared on a HOPG electrode by CV polymerization using potential scanning from -0.2 V to 0.78 V over 5 cycles of 0.2 mM of monomer **3** in 0.1 M $TBACF_3SO_3$/ACN electrolyte. Film thickness is 45 nm.

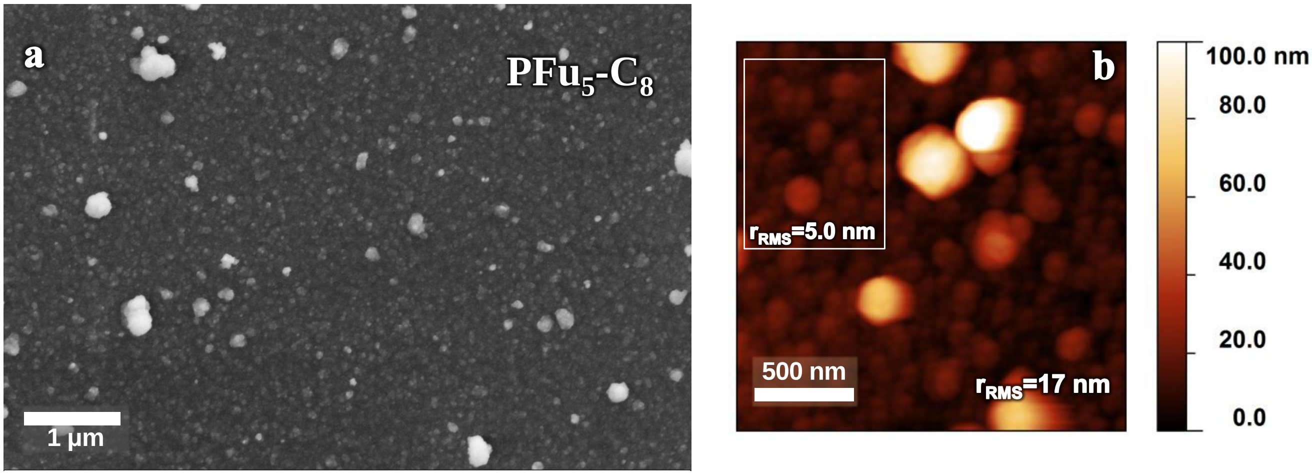

Figure S20. (a) SEM and (b) AFM images of **P3** (PFu_5-C_8) film prepared on a HOPG electrode by by CV polymerization using potential scanning from -0.2 V to 0.78 V over 10 cycles of 0.2 mM of monomer **3** in 0.1 M $TBACF_3SO_3$/ACN electrolyte. Film thickness is 90 nm. Total film area rms roughness is 17 nm, while that of the underlying film (white box area) is 5 nm.

With extended polymerization globular particles are observed on-top of the polymeric film.

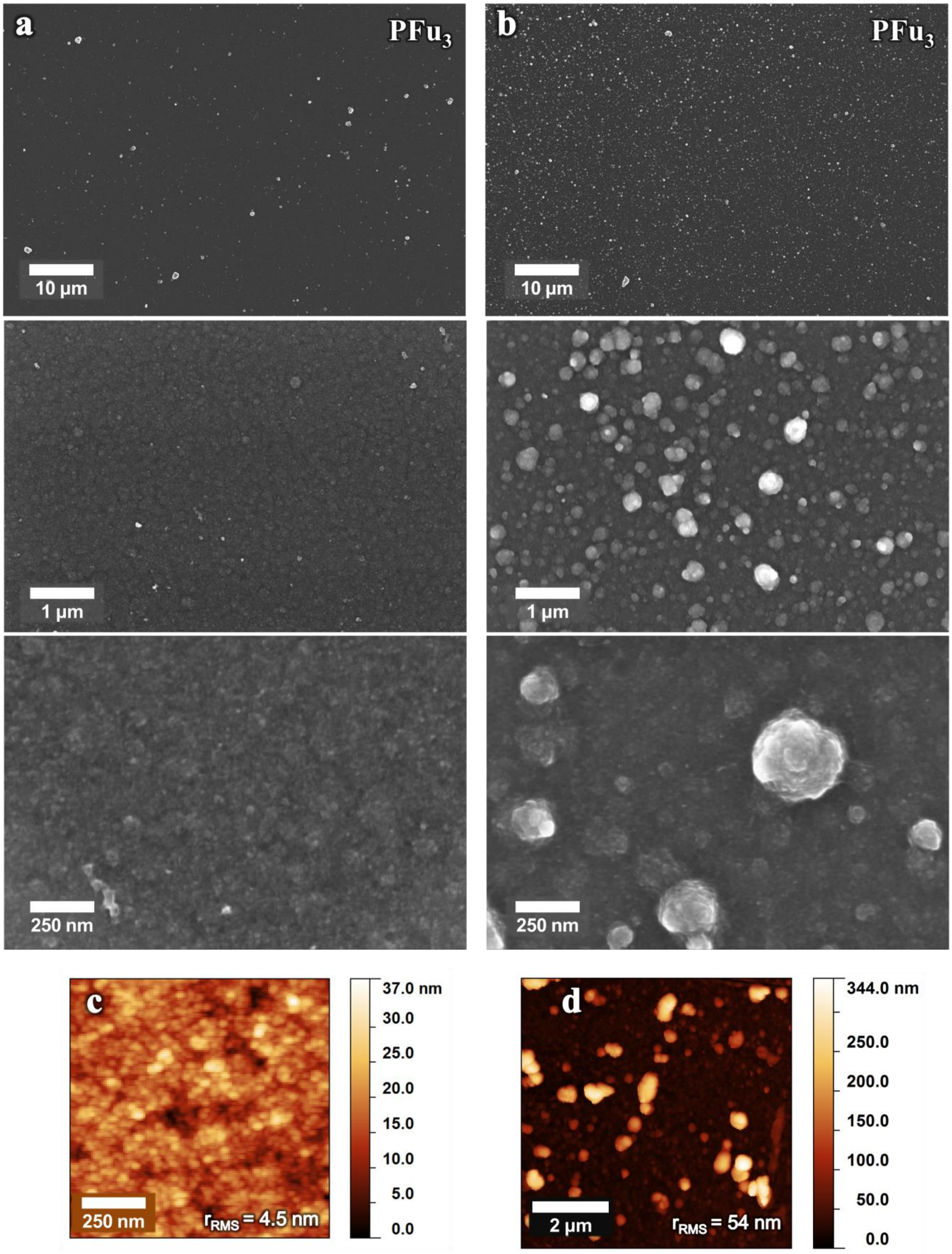

Figure S21. (a-b) SEM images and (c-d) AFM images of **P1** on an ITO electrode. The film thickness (not including on-top structures) of (a,c) is 50 nm and of (b,d) is 100 nm, as measured by AFM profilometry.

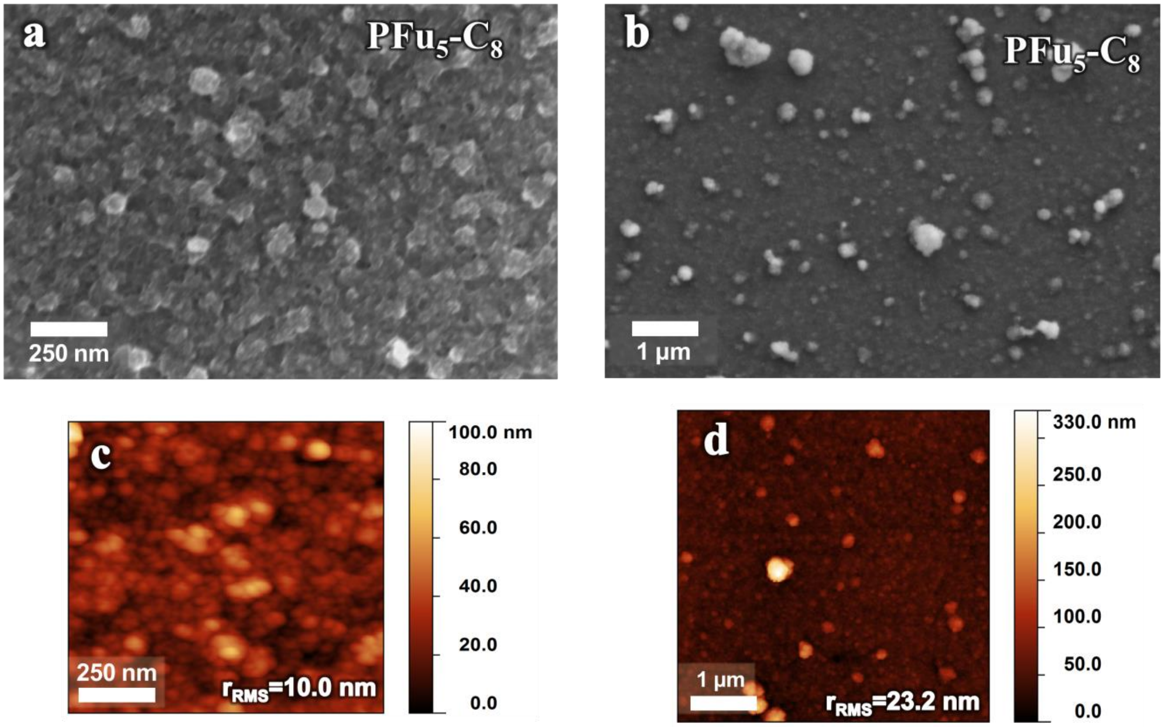

Figure S22. (a-b) SEM images and (c-d) AFM images of **P3** on an ITO electrode. The thickness of the film (a,c) is smaller than of (b,d).

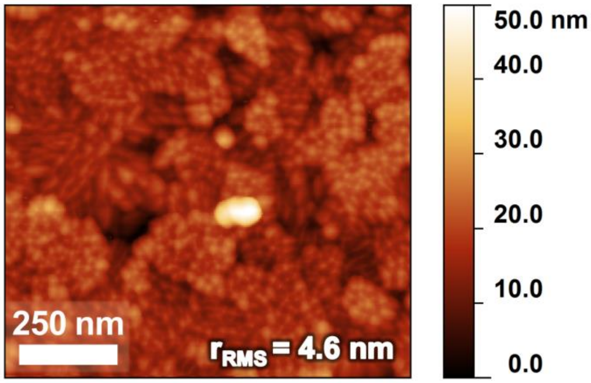

Figure S23. AFM image of clean ITO electrode.

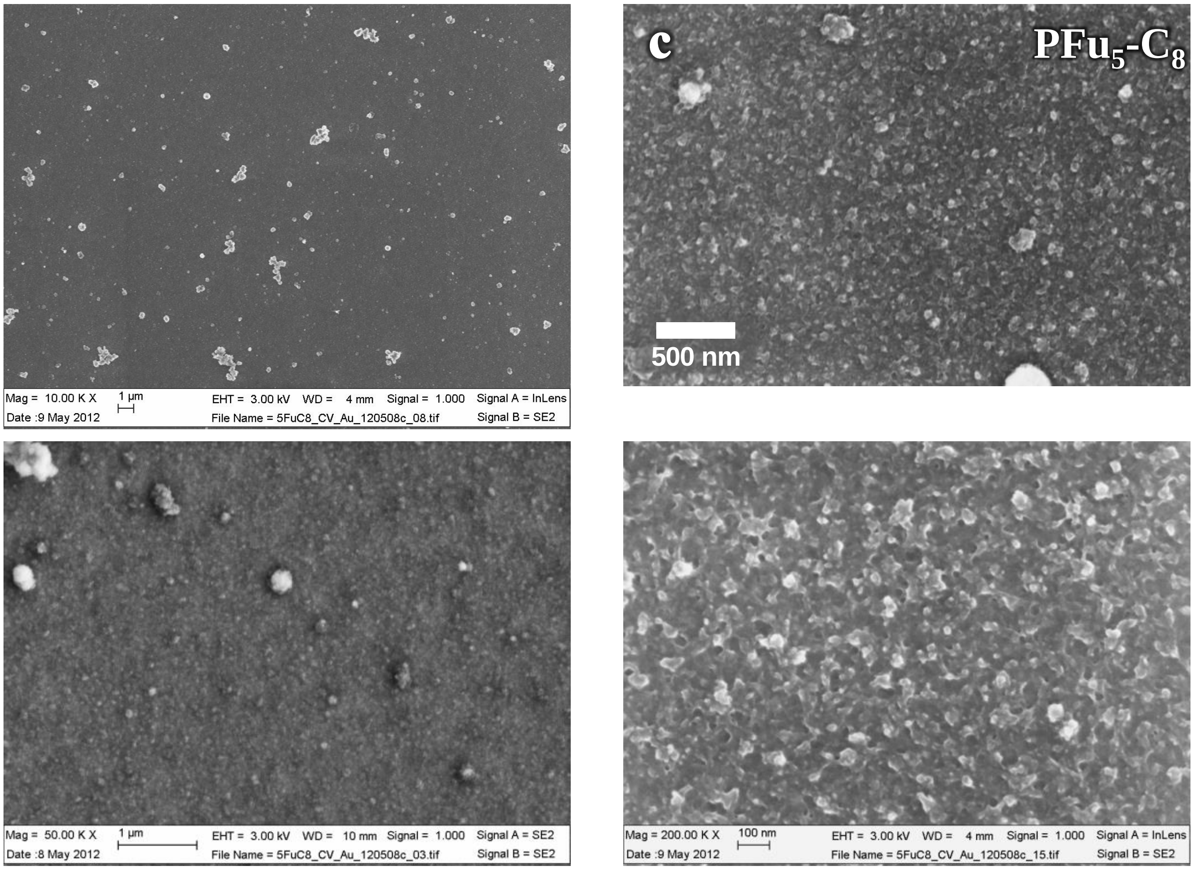

Figure S24. SEM images of **P3** (PFu_5-C_8) on an Au-coated glass electrode.

Estimation of polyfuran effective π-conjugation length from UV-Vis spectroscopy

The effective conjugation length in **P1** (λ_{max}=466 nm) is approximated to be about 33 furan units as deducted from second order polynomial extrapolation of absorption maxima of unsubstituted polyfurans as function of the reciprocal of the chain length. (Fig. S25a). Similarly, effective conjugation lengths in alkyl substituted polyfurans **P2-P7** (average λ_{max}=460 nm) is about 27 furan units based on extrapolation of λ_{max} of dihexyl substituted oligofurans (Fig. S25b). We note that such analysis could serve only an approximation to effective π-conjugation length.

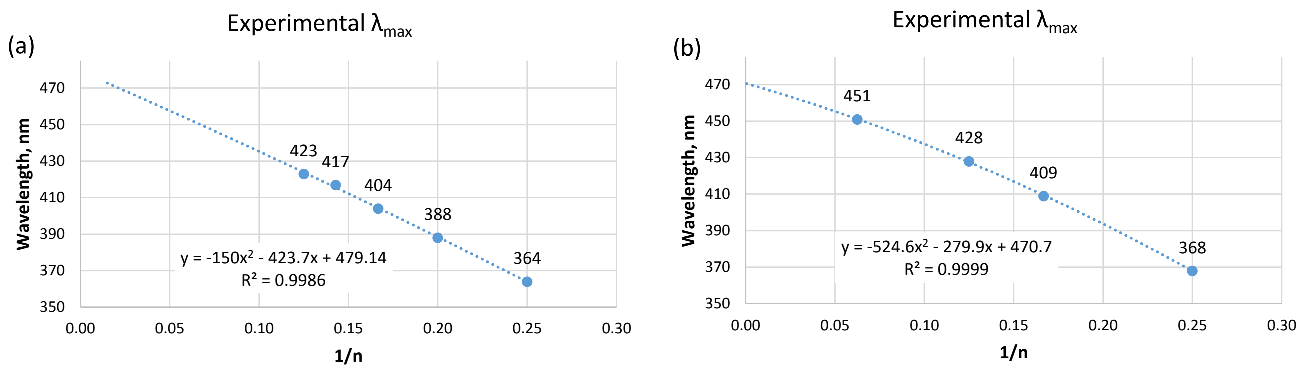

Figure S25. Second order polynomial fit of absorption maxima against the reciprocal of the number of monomer units (1/n) in (a) unsubstituted[1b] and (b) dihexyl substituted a-oligofurans.[14]

EQCM study of polyfurans

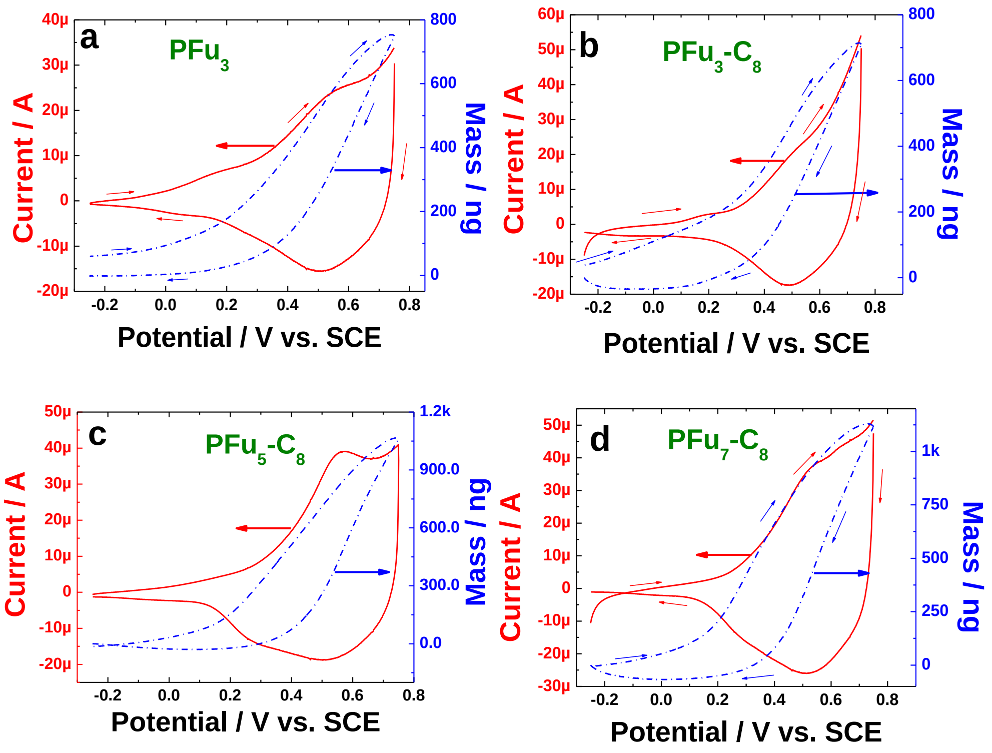

Figure S26. The EQCM-CV response of polymers (a) **P1**, (b) **P2**, (c) **P3** and (d) **P4** on a gold quartz crystal (Au/QC) electrode in ACN/0.1 M $TBACF_3SO_3$ solution at a sweep rate of 10 mV s^{-1}.

EQCM measurements were carried out in a Teflon cell consisting of an Au coated quartz crystal purchased from CH Instruments as the working electrode, a Pt-wire counter electrode, and a Ag/AgCl pseudo reference electrode. A quartz crystal of 13.7 mm diameter was AT-cut and sandwiched between 5.11 mm diameter vapor-deposited gold disks. The resonating frequency of the crystal in air was 8 MHz, and the surface area of the Au electrode was 0.205 cm^2. The shear modulus (μ) of the crystal was 2.947×10^{11} g $cm^{-1}s^{-2}$ and the density (ρ) was 2.648 g cm^{-3}. Before the electrochemical experiments, the working electrode was run in double distilled water to obtain a stable Δf frequency versus time plot. Subsequently, the double distilled water was replaced with the experimental electrolyte. The polymer films were deposited by cyclic voltammetry at a sweep rate of 50 mV s^{-1} in the potential range -0.2 to 0.8 V vs. Ag/AgCl-wire (pseudo reference) on Au quartz crystal. The deposition was stopped when the resonance frequency of the quartz crystal decreased by 3.5 kHz. After preparation, films were washed carefully with acetonitrile and cycled in monomer-free 0.1 M $TBACF_3SO_3$/ACN with a sweep rate of 50 mV s^{-1} between -0.8 to 0.8 V until stable voltammograms were obtained. The doping-dedoping behavior in acetonitrile with 0.1 M $TBACF_3SO_3$ was studied by CV at a sweep rate of 10 mV s^{-1} in the potential range of -0.2 to 0.8 V unless otherwise specified. Electrochemical studies were carried out using a computer-controlled CH Instruments potentiostat/galvanostat model 400 A. The software program provided electrochemical data in the form of voltammograms as well as the frequency of the crystal as a function of the potential. The data regarding changes in the frequency (Δf) of the crystal due to electrodeposits were converted into plots of mass changes (Δm) using the Sauerbrey relationship.

1. (a) H. Ishida, K. Yui, Y. Aso, T. Otsubo, F. Ogura, "Novel Electron Acceptors Bearing a Heteroquinonoid System III: 2,5-Bis(dicyanomethylene)-2,5-dihydrofuran and Its Conjugated Homologues as Novel Oxygen-Containing Electron Acceptors", *Bull. Chem. Soc. Jpn.* **1990**, *63*, 2828; (b) O. Gidron, Y. Diskin-Posner, M. Bendikov, "α-Oligofurans", *J. Am. Chem. Soc.* **2010**, *132*, 2148.

2. J. K. Politis, J. C. Nemes, M. D. Curtis, "Synthesis and Characterization of Regiorandom and Regioregular Poly(3-octylfuran)", *J. Am. Chem. Soc.* **2001**, *123*, 2537.

3. T. Kauffmann, B. Greving, R. Kriegesmann, A. Mitschker, A. Woltermann, "Heterocyclopolyaromaten, VI: Heterocyclotetraaromaten mit gleichartigen aromatischen Ringgliedern", *Chem. Ber.* **1978**, *111*, 1330.

4. Gaussian 09, Revision A.02, Frisch, M. J. Trucks, G. W. Schlegel, H. B. Scuseria, G. E. Robb, M. A. Cheeseman, J. R. Scalmani, G. Barone, V. Mennucci, B. Petersson, G. A. Nakatsuji, H. Caricato, M. Li, X. Hratchian, H. P. Izmaylov, A. F. Bloino, J. Zheng, G. Sonnenberg, J. L. Hada, M. Ehara, M. Toyota, K. Fukuda, R. Hasegawa, J. Ishida, M. Nakajima, T. Honda, Y. Kitao, O. Nakai H., Vreven, T. Montgomery, J. A. Peralta, Jr., J. E. Ogliaro, F. Bearpark, M. Heyd, J. J. Brothers, E. Kudin, K. N. Staroverov, V. N. Kobayashi, R. Normand, J. Raghavachari, K. Rendell, A. Burant, J. C. Iyengar, S. S. Tomasi, J. Cossi, M. Rega, N. Millam, J. M. Klene, M. Knox, J. E. Cross, J. B. Bakken, V. Adamo, C. Jaramillo, J. Gomperts, R. Stratmann, R. E. Yazyev, O. Austin, A. J. Cammi, R. Pomelli, C. Ochterski, J. W. Martin, R. L. Morokuma,. K. Zakrzewski, V. G Voth, G. A. Salvador, P. Dannenberg, J. J. Dapprich, S. Daniels, A. D. Farkas, Ö. Foresman, J. B. Ortiz, J. V. Cioslowski, J. Fox, D. J. Gaussian, Inc., Wallingford CT, 2009.

5. (a) Density-functional Theory of Atoms and Molecules, Parr, R. G.; Yang, W. Oxford University Press: New York, 1989. (b) A Chemist's Guide to Density Functional Theory; Koch, W.; Holthausen, M. C. Wiley-VCH: New York, 2000. (c) Lee, C.; Yang, W.; Parr, R. G. Phys. Rev. B 1988, 37, 785–789. (d) Becke, A. D. J. Chem. Phys. 1993, 98, 5648–5652.

6. (a) S. S. Zade, M. Bendikov, "From Oligomers to Polymer: Convergence in the HOMO−LUMO Gaps of Conjugated Oligomers", *Org. Lett.* **2006**, *8*, 5243; (b) S. S. Zade, N. Zamoshchik, M. Bendikov, "From Short Conjugated Oligomers to Conjugated Polymers. Lessons from Studies on Long Conjugated Oligomers", *Acc. Chem. Res.* **2011**, *44*, 14.

7. NIST Computational Chemistry Comparison and Benchmark Database, NIST Standard Reference Database Number 101, Release 15b, August 2011, Editor: Russell D. Johnson III, http://cccbdb.nist.gov/.

8. G. Zotti, G. Schiavon, "The polythiophene puzzle. Electrochemical and spectroelectrochemical evidence for two oxidation levels", *Synth. Met.* **1989**, *31*, 347.

9. G. Tourillon, F. Garnier, "New electrochemically generated organic conducting polymers", *J. Electroanal. Chem.* **1982**, *135*, 173.

10. T. Ohsawa, K. Kaneto, K. Yoshino, "Electrical and Optical Properties of Electrochemically Prepared Polyfuran", *Jpn. J. Appl. Phys.* **1984**, *23*, L663.

11. H. S. Nalwa, "Phase transitions in polypyrrole and polythiophene conducting polymers demonstrated by magnetic susceptibility measurements", *Phys. Rev. B* **1989**, *39*, 5964.

12. Nova, Version 1.0.26.1099. http://www.ntmdt.com/

13. Gwyddion, Version 2.26. http://gwyddion.net/

14. X.-H. Jin, D. Sheberla, L. J. W. Shimon, M. Bendikov, "Highly Coplanar Very Long Oligo(alkylfuran)s: A Conjugated System with Specific Head-To-Head Defect", *J. Am. Chem. Soc.* **2014**, *136*, 2592.
